# Causal association between solar ultraviolet exposure and vitiligo: a two-sample mendelian randomization study

**DOI:** 10.1007/s00403-025-03928-2

**Published:** 2025-02-13

**Authors:** Yuanyu Feng, Zhiwu Dong, Shenglan Wang, Lingshuang Li, Xiaoyan Yang, Yunmin Ma, Tianyu Li, Ruike Zhao, Haolei Wang, Dongjie Sun

**Affiliations:** 1 Department of Dermatology, The First Affiliated Hospital of Kunming Medical University, Kunming, Yunnan 650032 China; 2 Department of Urology, The First Affiliated Hospital of Kunming Medical University, Kunming, Yunnan 650032 China

To the editor,

Vitiligo is an autoimmune disease characterized by depigmented patches of skin caused by progressive loss of melanocytes [1]. Cutaneous lesions of vitiligo often surface at sun-exposed parts. Ultraviolet (UV) spanning wavelength range of 100–400 nm is mostly highlighted in the pathogenic effect of solar radiation. Previous observational studies indicated solar UV exposure may be a risk factor of vitiligo [2]. However, the casual association between solar UV exposure and vitiligo remains unclear. Mendelian randomization (MR) is a statistical method using genetic variants as instrumental variables (IVs) to evaluate the causal relationship between exposures and outcomes. Presently, we performed a two-sample MR analysis to investigate the casual effect of solar UV exposure-triggered cutaneous responses including sunburn, childhood sunburn occasions, and ease of skin tanning on vitiligo.

Figure 1 illustrates our study design. Data sources and IVs applied were detailed in supplementary materials. Notably, two different summary GWAS data of vitiligo were used as the main and validation analysis respectively. Inverse variance weighted (IVW) method was utilized as the main approach supplemented by the weighted median and MR-Egger methods. Heterogeneity was tested by Cochran’s Q test. Directional pleiotropy was evaluated by MR-Egger intercept test and MR-PRESSO global test. After the Benjamini-Hochberg method correction, *q value < 0.05* was considered significant while *p value < 0.05* but *q value > 0.05* indicated suggestive association.

**Fig. 1:**
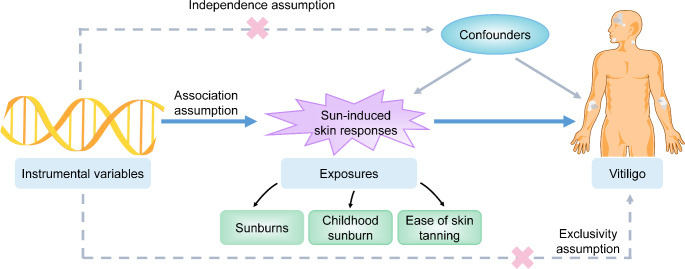
Design and assumption of Mendelian randomization.

Our results indicated that sunburn (IVW: OR = 0.179; 95% CI, 0.049–0.656; *p* = 0.009; *q < 0.05*), childhood sunburn (IVW: OR = 0.361; 95% CI, 0.162–0.805; *p* = 0.013; *q < 0.05*), and ease of skin tanning (IVW: OR = 0.684; 95% CI, 0.482–0.970; *p* = 0.033; *q < 0.05*) were significantly associated with the decreased risk of vitiligo (Fig. 2), which was supported by the results of validation MR analysis (Fig. S2). Cochran’s Q test suggested no heterogeneity except childhood sunburn occasions in the main analysis and ease of skin tanning in the validation analysis. This problem was addressed by using the random-effects IVW method. Meanwhile, no horizontal pleiotropy was found in our study. Leave-one-out analysis showed the unaffected causal estimates (supplementary materials).

**Fig. 2:**
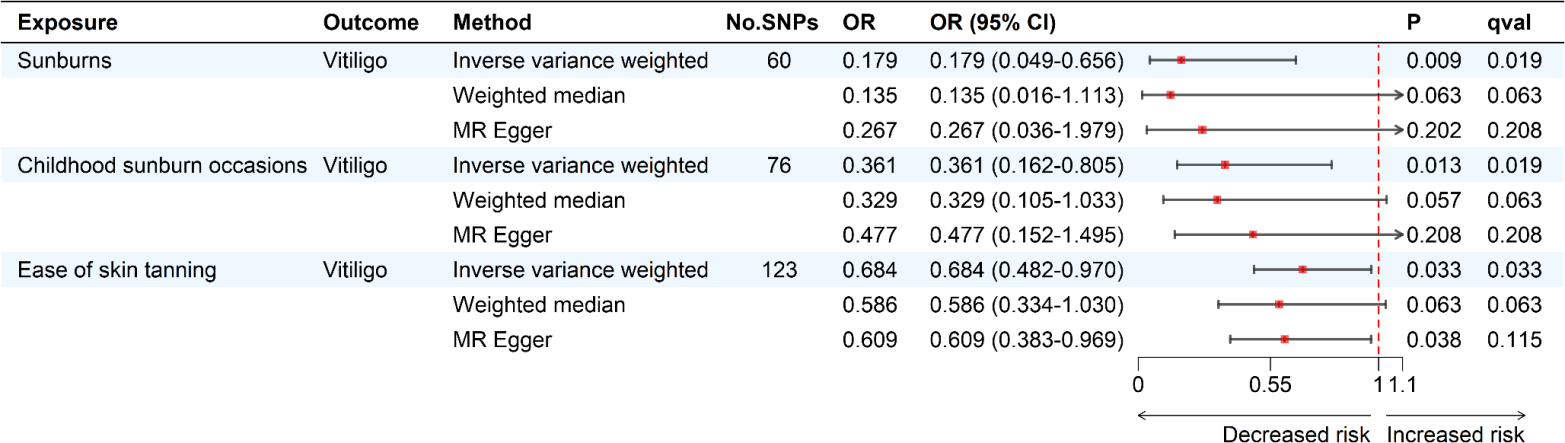
Forest plot of the main MR analysis. After the Benjamini-Hochberg method correction, *q < 0.05* was considered significant. *p value < 0.05* but *q value > 0.05* indicated a suggestive association. Note: CI, confidence interval; MR, Mendelian randomization; OR, odds ratio; SNP, single nucleotide polymorphisms.

In conclusion, our study indicated that sunburn, childhood sunburn, and ease of skin tanning may reduce the risk of vitiligo in the European population, providing novel insights into the pathogenesis and prevention of vitiligo. Notably, this conclusion differs from previous observational studies, which may be attributed to the discrepancy between observational studies and MR research. Solar UV radiation can upregulate programmed death-ligand 1 to mediate immunosuppression or induce regulatory T cells to maintain immunological tolerance and avoid autoimmune disorders [1, 3]. Furthermore, it facilitates melanin genesis by directly activating the melanocytes or by inducing the production of immune mediators such as prostaglandin E2 and vitamin D [4]. These may be the plausible mechanisms of the protective effect of solar UV radiation on vitiligo [5]. However, current available GWAS data limited further investigations including generalization of our findings to other populations and sub-analysis stratified by sex. Further research is required to explore the potential mechanisms.

## Electronic supplementary material

Below is the link to the electronic supplementary material.

Supplementary Material 1

## Data Availability

The data were publicly accessible, and data generated or analyzed during this study are provided in this article or in the supplementary material files.

## Abbreviations

IVs: Instrumental variables
IVW: Inverse variance weighted
MR: Mendelian randomization
MR-PRESSO: Mendelian Randomization Pleiotropy Residual Sum and Outlier
UV: Utraviolet
