# Supplementary material for: Causal association between solar ultraviolet exposure and vitiligo: a two-sample mendelian randomization study
Source: Arch Dermatol Res. 2025 Feb 13;317(1):397. doi: 10.1007/s00403-025-03928-2 (PMC11825587; doi:10.1007/s00403-025-03928-2)
Supplement: Supplementary file 1 — Supplementary Material 1 [file 403_2025_3928_MOESM1_ESM.docx]

**Causal association between solar ultraviolet exposure and vitiligo: a two-sample** **Mendelian randomization study**

Yuanyu Feng^1^, Zhiwu Dong^2^, Shenglan Wang^1^, Lingshuang Li^1^, Xiaoyan Yang^1^,

Yunmin Ma^1^, Tianyu Li^1^, Ruike Zhao^1^, Haolei Wang^1^, Dongjie Sun^1^*

1. Department of Dermatology, The First Affiliated Hospital of Kunming Medical University, Kunming, Yunnan 650032, China

2. Department of Urology, The First Affiliated Hospital of Kunming Medical University, Kunming, Yunnan 650032, China.

***Correspondence:**

DongJie Sun

E-mail: sundongjieder@126.com

Figure S1

**
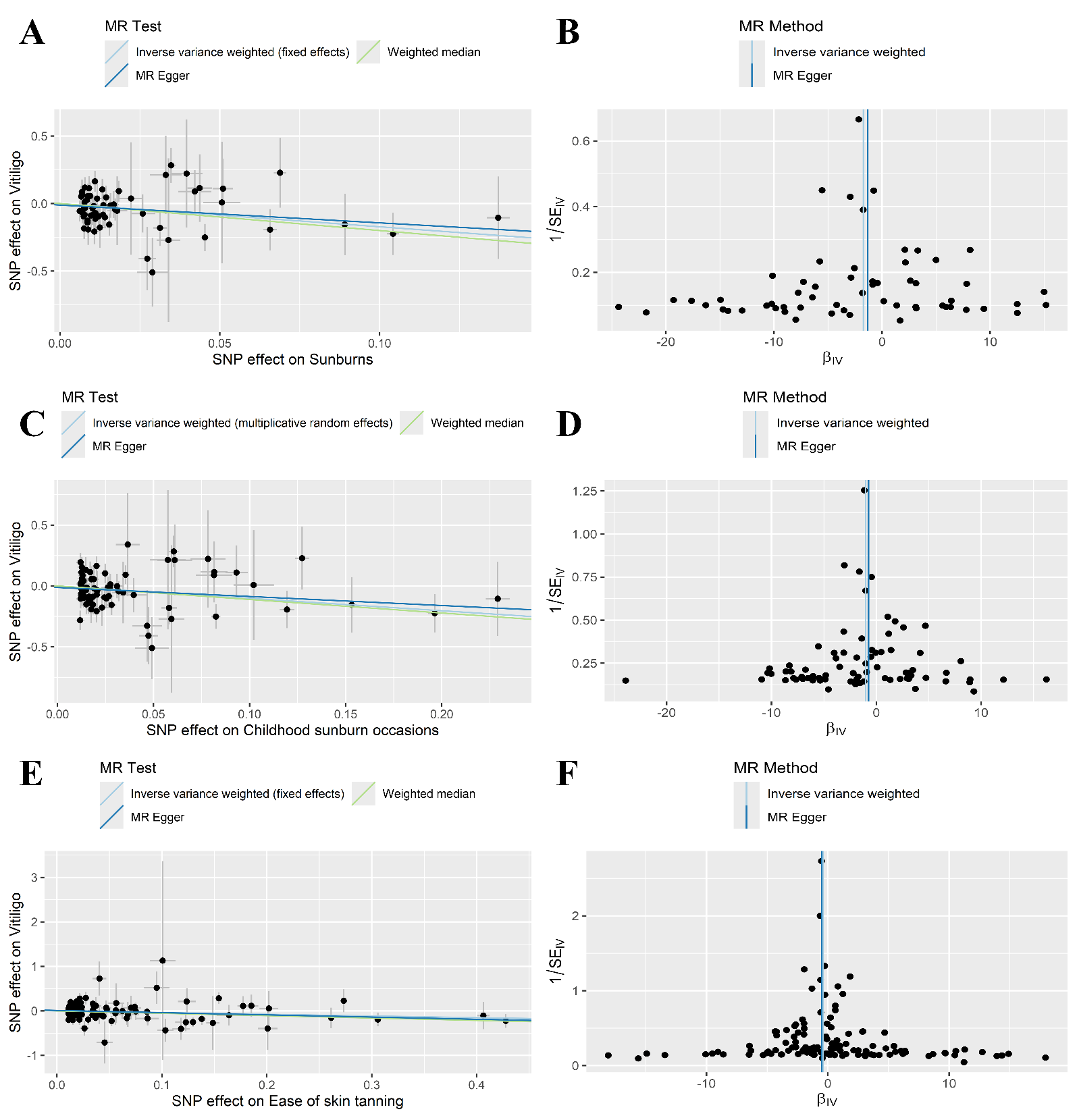
**

**Figure S1.** Scatter and funnel plots of causal relationship between UV and vitiligo in main MR results. (A) Scatter plot of sunburns on vitiligo；(B) Funnel plot of sunburns on vitiligo; (C) Scatter plot of childhood sunburn occasions on vitiligo；(D) Funnel plot of childhood sunburn occasions on vitiligo; (E) Scatter plot of ease of skin tanning on vitiligo；(F) Funnel plot of ease of skin tanning on vitiligo. Note: MR, Mendelian randomization; SNP, Single nucleotide polymorphisms; UV, ultraviolet.

Figure S2

**
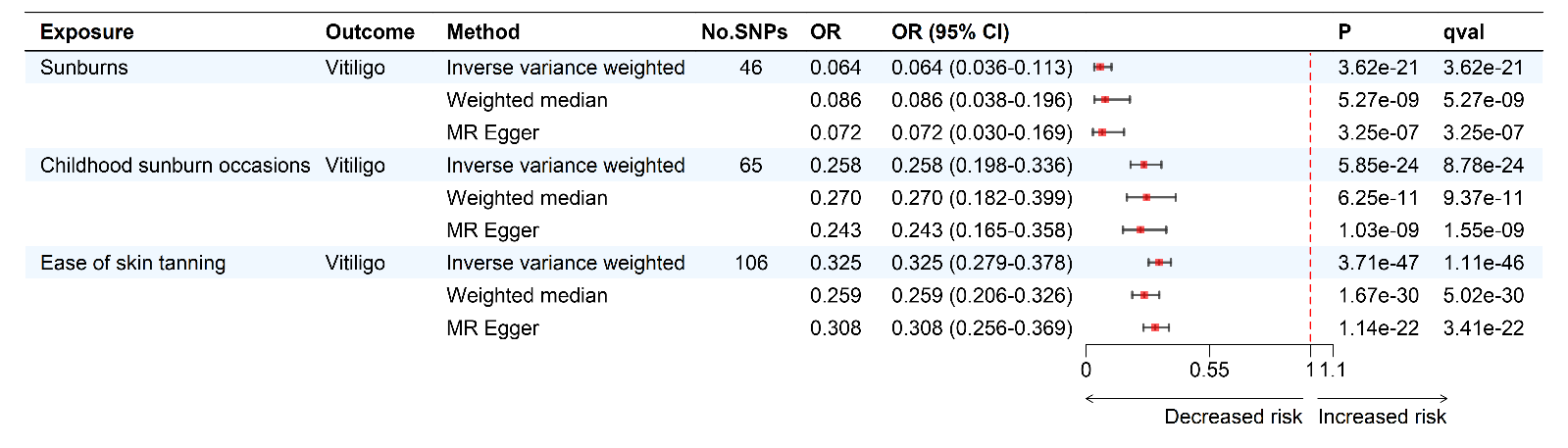
**

**Figure S2.** Forest plot of the validation MR analysis. After the Benjamini-Hochberg method correction, *q value < 0.05* was considered significant while *p value < 0.05* but *q value > 0.05* indicated suggestive association. Note: CI, confidence interval; MR, Mendelian randomization; OR, odds ratio; SNP, Single nucleotide polymorphisms.

Figure S3

**
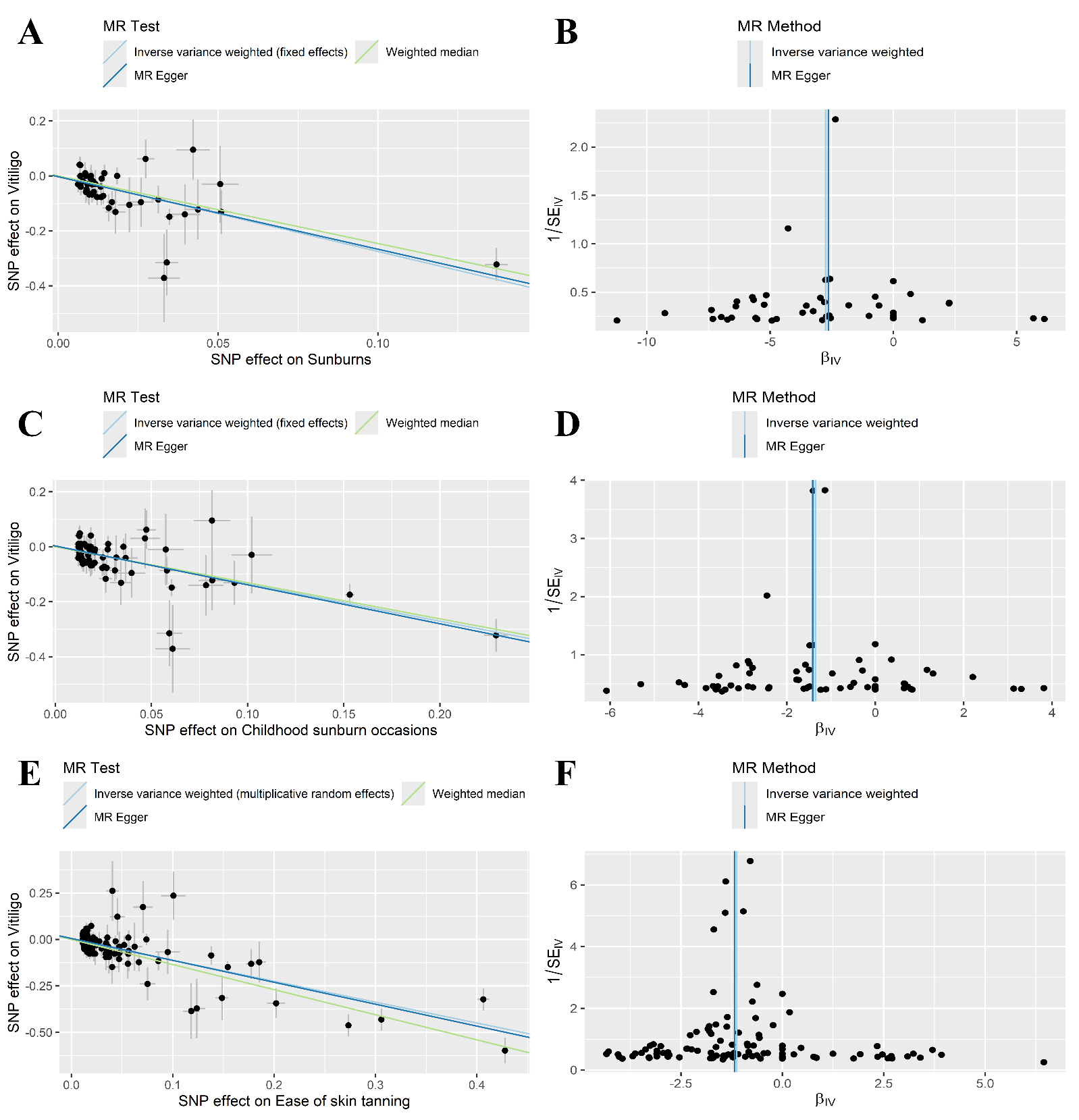
**

**Figure S3.** Scatter and funnel plots of validation MR results. (A) Scatter plot of sunburns on vitiligo；(B) Funnel plot of sunburns on vitiligo; (C) Scatter plot of childhood sunburn occasions on vitiligo；(D) Funnel plot of childhood sunburn occasions on vitiligo; (E) Scatter plot of ease of skin tanning on vitiligo；(F) Funnel plot of ease of skin tanning on vitiligo. Note: MR, Mendelian randomization; SNP, Single nucleotide polymorphisms; UV, ultraviolet.

Figure S4

**
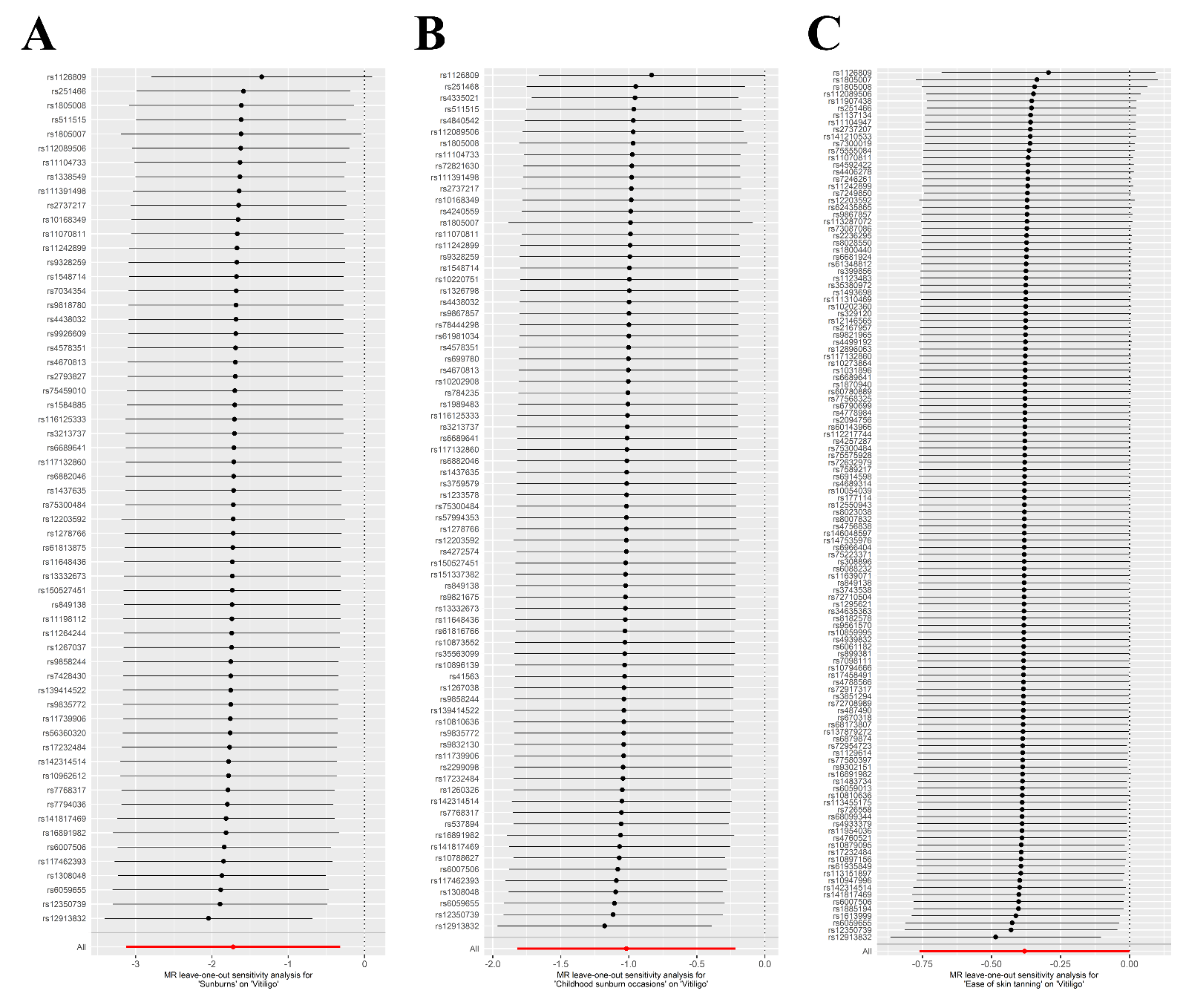
**

**Figure S4.** Leave-one-out plots for the genetic association between solar UV-induced cutaneous responses and risk of vitiligo in the main MR analysis. (A) Sunburns on vitiligo；(B) Childhood sunburn occasions on vitiligo; (C) Ease of skin tanning on vitiligo. Note: MR, Mendelian randomization; UV, ultraviolet.

Figure S5


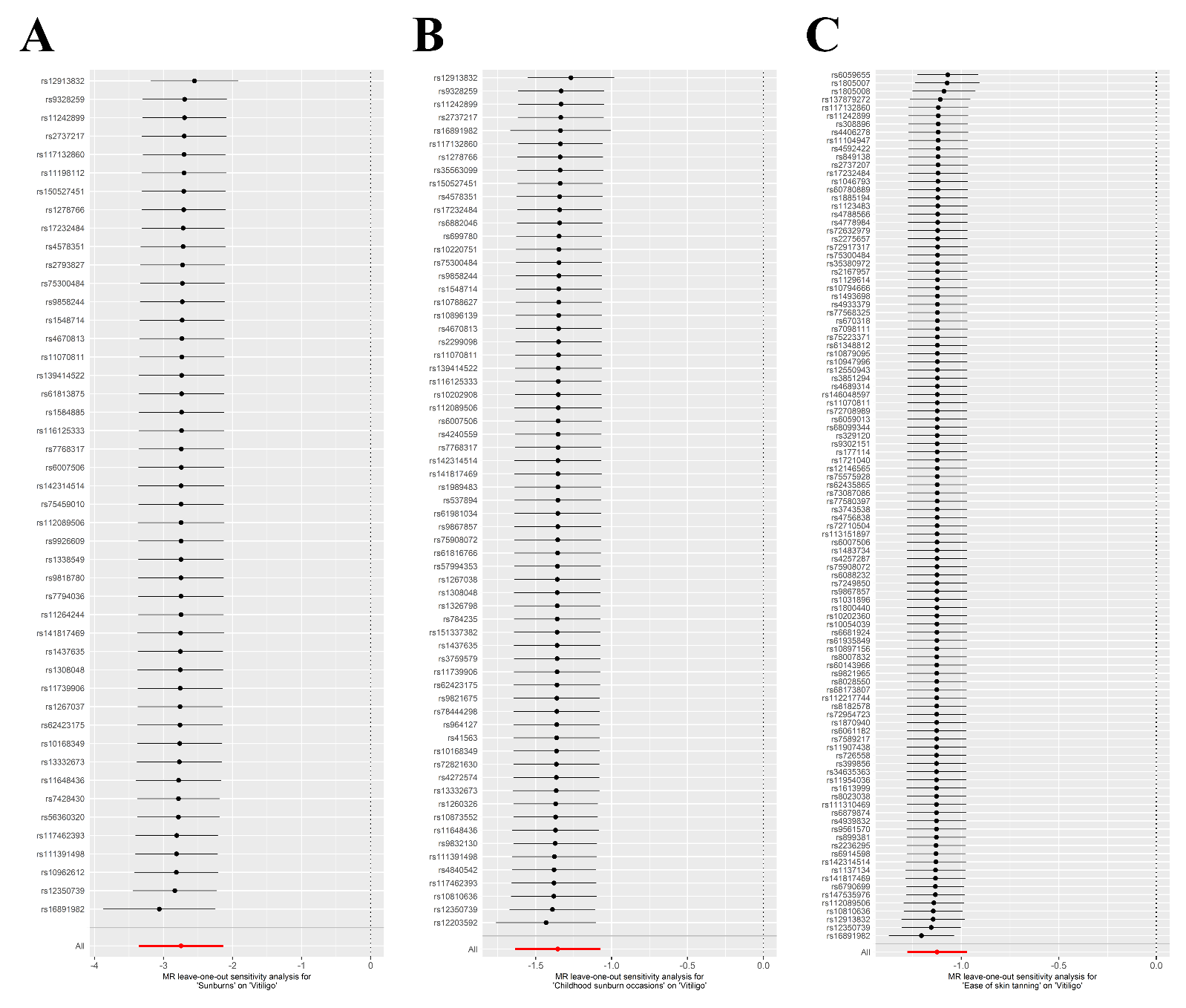


**Figure S4.** Leave-one-out plots for the genetic association between solar UV-induced cutaneous responses and risk of vitiligo in the validation MR analysis. (A) Sunburns on vitiligo；(B) Childhood sunburn occasions on vitiligo; (C) Ease of skin tanning on vitiligo. Note: MR, Mendelian randomization; UV, ultraviolet.

**Table S1.** Summary information of data sources.

| Trait | GWAS ID | Year | Sample size | Ancestry |
| --- | --- | --- | --- | --- |
| Vitiligo (main analysis) | finngen_R11_L12_VITILIGO | 2024 | 423380 | European |
| Vitiligo (validation analysis) | GCST004785 | 2016 | 44266 | European |
| Sunburns | ebi-a-GCST90029034 | 2018 | 350232 | European |
| Childhood sunburn occasions | ukb-b-13246 | 2018 | 346,955 | European |
| Ease of skin tanning | ukb-b-533 | 2018 | 453065 | European |

**Table S2.** The results of the sensitivity analyses and MR-Steiger directional test in the main MR analysis.

| Exposure | Outcome |  | Cochran' Q Test | | |  | MR-Egger Test | | |  | MR-PRESSO test | |  | Steiger test |
| --- | --- | --- | --- | --- | --- | --- | --- | --- | --- | --- | --- | --- | --- | --- |
|  |  |  | method | Q | p-value |  | Egger intercept | SE | p-value |  | p-value | Outliers |  | Causal direction |
| Sunburns | Vitiligo |  | Inverse variance weighted | 68.712 | 0.181 |  | -0.011 | 0.020 | 0.580 |  | 0.171 | NA |  | TRUE |
| Childhood sunburn occasions | Vitiligo |  | Inverse variance weighted | 100.156 | 0.028 |  | -0.012 | 0.019 | 0.506 |  | 0.057 | NA |  | TRUE |
| Ease of skin tanning | Vitiligo |  | Inverse variance weighted | 144.899 | 0.077 |  | 0.010 | 0.012 | 0.392 |  | 0.079 | NA |  | TRUE |

MR, Mendelian Randomization; NA, not available; Q, Cochran’s Q statistic; SE, standard error.

**Table S3.** The results of the sensitivity analyses and MR-Steiger directional test in the validation MR analysis.

| Exposure | Outcome |  | Cochran' Q Test | | |  | MR-Egger Test | | |  | MR-PRESSO test | |  | Steiger test |
| --- | --- | --- | --- | --- | --- | --- | --- | --- | --- | --- | --- | --- | --- | --- |
|  |  |  | method | Q | p-value |  | Egger intercept | SE | p-value |  | p-value | Outliers |  | Causal direction |
| Sunburns | Vitiligo |  | Inverse variance weighted | 51.515 | 0.234 |  | -0.003 | 0.008 | 0.715 |  | 0.251 | NA |  | TRUE |
| Childhood sunburn occasions | Vitiligo |  | Inverse variance weighted | 73.733 | 0.190 |  | 0.003 | 0.007 | 0.651 |  | 0.220 | NA |  | TRUE |
| Ease of skin tanning | Vitiligo |  | Inverse variance weighted | 150.175 | 0.003 |  | 0.005 | 0.005 | 0.294 |  | 0.053 | NA |  | TRUE |

MR, Mendelian Randomization; NA, not available; Q, Cochran’s

**Table S4.** Instrumental variables used in main analysis.

| SNP | Effect allele | Other allele | beta.exposure | se.exposure | eaf.exposure | pval.exposure | beta.outcome | se.outcome | eaf.outcome | pval.outcome | Steiger  direction | R^2^ | F |
| --- | --- | --- | --- | --- | --- | --- | --- | --- | --- | --- | --- | --- | --- |
| Sunburns on vitiligo (main analysis) | | | | | | | | | | | | | |
| rs10168349 | C | G | 0.008641 | 0.001233 | 0.335454 | 1.20E-13 | -0.140949 | 0.086224 | 0.280817 | 1.02E-01 | TRUE | 1.40E-04 | 49.100 |
| rs10962612 | G | T | 0.014426 | 0.001365 | 0.761024 | 6.30E-26 | 0.045273 | 0.086511 | 0.727016 | 6.01E-01 | TRUE | 3.19E-04 | 111.708 |
| rs11070811 | T | C | -0.008823 | 0.001504 | 0.182873 | 5.10E-09 | 0.192501 | 0.113156 | 0.133263 | 8.89E-02 | TRUE | 9.82E-05 | 34.413 |
| rs11104733 | T | C | 0.028916 | 0.004841 | 0.014726 | 2.40E-09 | -0.509677 | 0.254882 | 0.023720 | 4.55E-02 | TRUE | 1.02E-04 | 35.681 |
| rs111391498 | G | A | -0.027331 | 0.002717 | 0.048094 | 6.90E-24 | 0.408525 | 0.234693 | 0.028378 | 8.17E-02 | TRUE | 2.89E-04 | 101.204 |
| rs11198112 | T | C | -0.016809 | 0.001589 | 0.165023 | 2.30E-26 | 0.007133 | 0.100297 | 0.183877 | 9.43E-01 | TRUE | 3.19E-04 | 111.840 |
| rs112089506 | T | C | -0.031302 | 0.002183 | 0.081295 | 2.00E-47 | 0.180376 | 0.134048 | 0.094132 | 1.78E-01 | TRUE | 5.87E-04 | 205.554 |
| rs11242899 | A | G | -0.012129 | 0.001319 | 0.266336 | 1.10E-19 | 0.094052 | 0.087927 | 0.261521 | 2.85E-01 | TRUE | 2.42E-04 | 84.610 |
| rs11264244 | T | C | -0.011619 | 0.002103 | 0.085994 | 2.90E-08 | -0.036753 | 0.128268 | 0.100769 | 7.74E-01 | TRUE | 8.72E-05 | 30.533 |
| rs1126809 | A | G | 0.045350 | 0.001268 | 0.303381 | 1.00E-200 | -0.251597 | 0.100794 | 0.177232 | 1.26E-02 | TRUE | 3.64E-03 | 1279.055 |
| rs116125333 | G | T | -0.025883 | 0.003934 | 0.023117 | 2.50E-10 | 0.074232 | 0.140560 | 0.083237 | 5.97E-01 | TRUE | 1.24E-04 | 43.295 |
| rs11648436 | T | C | -0.013577 | 0.001209 | 0.356060 | 7.90E-31 | 0.011707 | 0.083466 | 0.307005 | 8.88E-01 | TRUE | 3.60E-04 | 126.056 |
| rs117132860 | A | G | 0.033950 | 0.003682 | 0.025556 | 6.30E-21 | -0.271036 | 0.606270 | 0.004001 | 6.55E-01 | TRUE | 2.43E-04 | 85.016 |
| rs11739906 | C | A | 0.006903 | 0.001239 | 0.327118 | 2.70E-08 | 0.086440 | 0.090270 | 0.240092 | 3.38E-01 | TRUE | 8.87E-05 | 31.057 |
| rs117462393 | T | C | 0.042182 | 0.005196 | 0.014415 | 5.70E-16 | 0.089137 | 0.156909 | 0.065008 | 5.70E-01 | TRUE | 1.88E-04 | 65.896 |
| rs12203592 | T | C | 0.089138 | 0.001436 | 0.219700 | 1.00E-200 | -0.155164 | 0.228257 | 0.030371 | 4.97E-01 | TRUE | 1.09E-02 | 3854.113 |
| rs12350739 | A | G | 0.018407 | 0.001200 | 0.607745 | 8.30E-55 | 0.091953 | 0.077416 | 0.543926 | 2.35E-01 | TRUE | 6.71E-04 | 235.275 |
| rs1267037 | C | A | -0.008423 | 0.001485 | 0.806239 | 4.10E-08 | -0.026294 | 0.088672 | 0.744253 | 7.67E-01 | TRUE | 9.19E-05 | 32.176 |
| rs1278766 | C | T | 0.010587 | 0.001170 | 0.546111 | 2.20E-20 | -0.019147 | 0.077117 | 0.486703 | 8.04E-01 | TRUE | 2.34E-04 | 81.853 |
| rs12913832 | G | A | 0.034765 | 0.001405 | 0.777588 | 1.50E-138 | 0.283293 | 0.129618 | 0.895544 | 2.88E-02 | TRUE | 1.75E-03 | 612.559 |
| rs1308048 | C | T | -0.010936 | 0.001183 | 0.419442 | 1.00E-19 | -0.164059 | 0.077709 | 0.429769 | 3.48E-02 | TRUE | 2.44E-04 | 85.425 |
| rs13332673 | T | G | -0.050659 | 0.005793 | 0.010085 | 1.20E-18 | -0.008078 | 0.450346 | 0.007522 | 9.86E-01 | TRUE | 2.18E-04 | 76.465 |
| rs1338549 | G | T | -0.007553 | 0.001168 | 0.532670 | 4.40E-11 | 0.184102 | 0.079719 | 0.627355 | 2.09E-02 | TRUE | 1.19E-04 | 41.835 |
| rs139414522 | C | T | -0.039621 | 0.004940 | 0.015298 | 5.30E-16 | -0.222158 | 0.400716 | 0.009396 | 5.79E-01 | TRUE | 1.84E-04 | 64.339 |
| rs141817469 | T | C | -0.050991 | 0.003170 | 0.035111 | 2.50E-58 | -0.110242 | 0.221606 | 0.031490 | 6.19E-01 | TRUE | 7.38E-04 | 258.703 |
| rs142314514 | G | A | -0.043698 | 0.003824 | 0.028577 | 8.00E-31 | -0.114638 | 0.250202 | 0.029766 | 6.47E-01 | TRUE | 3.73E-04 | 130.597 |
| rs1437635 | A | C | 0.010176 | 0.001569 | 0.165792 | 3.60E-11 | -0.035871 | 0.120568 | 0.115232 | 7.66E-01 | TRUE | 1.20E-04 | 42.089 |
| rs150527451 | A | G | 0.015792 | 0.001896 | 0.106533 | 2.60E-16 | -0.013814 | 0.091549 | 0.233292 | 8.80E-01 | TRUE | 1.98E-04 | 69.340 |
| rs1548714 | C | A | 0.008660 | 0.001490 | 0.811844 | 8.80E-09 | -0.127519 | 0.099009 | 0.812556 | 1.98E-01 | TRUE | 9.65E-05 | 33.789 |
| rs1584885 | T | G | -0.006184 | 0.001188 | 0.603707 | 3.40E-08 | 0.055593 | 0.077256 | 0.489215 | 4.72E-01 | TRUE | 7.73E-05 | 27.088 |
| rs16891982 | G | C | 0.137113 | 0.003584 | 0.972918 | 1.00E-200 | -0.105041 | 0.305363 | 0.982617 | 7.31E-01 | TRUE | 4.16E-03 | 1463.849 |
| rs17232484 | A | G | 0.033094 | 0.004955 | 0.014140 | 4.30E-12 | 0.212095 | 0.290802 | 0.018867 | 4.66E-01 | TRUE | 1.27E-04 | 44.603 |
| rs1805007 | T | C | 0.104198 | 0.001908 | 0.101395 | 1.00E-200 | -0.224255 | 0.156542 | 0.066054 | 1.52E-01 | TRUE | 8.45E-03 | 2983.607 |
| rs1805008 | T | C | 0.065733 | 0.002051 | 0.086887 | 1.00E-200 | -0.193515 | 0.152876 | 0.068151 | 2.06E-01 | TRUE | 2.92E-03 | 1027.014 |
| rs251466 | G | C | -0.015405 | 0.001352 | 0.248444 | 5.90E-31 | 0.156125 | 0.081159 | 0.345025 | 5.44E-02 | TRUE | 3.70E-04 | 129.807 |
| rs2737217 | G | A | -0.014070 | 0.001180 | 0.562872 | 1.60E-33 | 0.102291 | 0.082192 | 0.665199 | 2.13E-01 | TRUE | 4.06E-04 | 142.173 |
| rs2793827 | T | A | -0.009696 | 0.001750 | 0.126104 | 3.60E-08 | 0.088160 | 0.102792 | 0.168185 | 3.91E-01 | TRUE | 8.76E-05 | 30.697 |
| rs3213737 | A | G | -0.017272 | 0.001183 | 0.576410 | 8.50E-50 | 0.044151 | 0.081081 | 0.655436 | 5.86E-01 | TRUE | 6.08E-04 | 213.096 |
| rs4438032 | G | C | 0.012466 | 0.001996 | 0.906538 | 1.40E-10 | -0.177765 | 0.150350 | 0.927912 | 2.37E-01 | TRUE | 1.11E-04 | 38.989 |
| rs4578351 | C | T | -0.011117 | 0.001409 | 0.221479 | 2.70E-15 | 0.071568 | 0.089639 | 0.247812 | 4.25E-01 | TRUE | 1.78E-04 | 62.279 |
| rs4670813 | A | G | -0.007021 | 0.001172 | 0.470742 | 7.70E-09 | 0.069095 | 0.077370 | 0.524672 | 3.72E-01 | TRUE | 1.02E-04 | 35.886 |
| rs511515 | G | A | -0.010761 | 0.001289 | 0.700735 | 3.80E-17 | 0.207585 | 0.092914 | 0.781183 | 2.55E-02 | TRUE | 1.99E-04 | 69.706 |
| rs56360320 | C | A | -0.006904 | 0.001174 | 0.566674 | 1.40E-09 | -0.065100 | 0.077467 | 0.546919 | 4.01E-01 | TRUE | 9.87E-05 | 34.575 |
| rs6007506 | T | C | -0.013253 | 0.001235 | 0.337412 | 7.10E-27 | -0.103816 | 0.080213 | 0.356734 | 1.96E-01 | TRUE | 3.29E-04 | 115.250 |
| rs6059655 | G | A | -0.068856 | 0.001963 | 0.897557 | 1.00E-200 | -0.227909 | 0.258239 | 0.977546 | 3.77E-01 | TRUE | 3.50E-03 | 1230.704 |
| rs61813875 | G | C | 0.022237 | 0.003591 | 0.030206 | 5.60E-11 | 0.037207 | 0.417163 | 0.008307 | 9.29E-01 | TRUE | 1.09E-04 | 38.347 |
| rs6689641 | G | A | 0.007843 | 0.001168 | 0.542835 | 7.50E-12 | -0.032929 | 0.077671 | 0.560178 | 6.72E-01 | TRUE | 1.29E-04 | 45.082 |
| rs6882046 | G | A | 0.007509 | 0.001327 | 0.267234 | 2.00E-09 | -0.035006 | 0.100295 | 0.180398 | 7.27E-01 | TRUE | 9.15E-05 | 32.037 |
| rs7034354 | G | A | 0.011075 | 0.001673 | 0.857656 | 1.10E-10 | -0.113129 | 0.106251 | 0.845091 | 2.87E-01 | TRUE | 1.25E-04 | 43.844 |
| rs7428430 | T | C | 0.006666 | 0.001167 | 0.487613 | 1.40E-08 | 0.051974 | 0.077498 | 0.547126 | 5.02E-01 | TRUE | 9.32E-05 | 32.631 |
| rs75300484 | T | C | -0.017894 | 0.003157 | 0.034802 | 1.20E-08 | 0.053386 | 0.254005 | 0.023242 | 8.34E-01 | TRUE | 9.17E-05 | 32.132 |
| rs75459010 | T | C | 0.010577 | 0.001864 | 0.115487 | 2.90E-08 | -0.079764 | 0.113917 | 0.134813 | 4.84E-01 | TRUE | 9.19E-05 | 32.185 |
| rs7768317 | T | C | -0.009092 | 0.001347 | 0.249001 | 2.80E-11 | -0.113762 | 0.087785 | 0.261317 | 1.95E-01 | TRUE | 1.30E-04 | 45.533 |
| rs7794036 | C | T | -0.007779 | 0.001165 | 0.530078 | 9.10E-11 | -0.118062 | 0.077309 | 0.537271 | 1.27E-01 | TRUE | 1.27E-04 | 44.551 |
| rs849138 | A | G | 0.007697 | 0.001165 | 0.507332 | 1.90E-10 | 0.010458 | 0.077279 | 0.492330 | 8.92E-01 | TRUE | 1.25E-04 | 43.671 |
| rs9328259 | A | C | 0.013462 | 0.001300 | 0.718160 | 1.80E-25 | -0.083084 | 0.086127 | 0.721066 | 3.35E-01 | TRUE | 3.06E-04 | 107.223 |
| rs9818780 | C | T | 0.007689 | 0.001168 | 0.489340 | 2.70E-11 | -0.082108 | 0.077945 | 0.429520 | 2.92E-01 | TRUE | 1.24E-04 | 43.359 |
| rs9835772 | T | A | 0.008667 | 0.001355 | 0.243743 | 1.30E-10 | 0.054960 | 0.091535 | 0.229146 | 5.48E-01 | TRUE | 1.17E-04 | 40.915 |
| rs9858244 | A | G | 0.009432 | 0.001421 | 0.214110 | 4.60E-11 | 0.055900 | 0.099321 | 0.184634 | 5.74E-01 | TRUE | 1.26E-04 | 44.068 |
| rs9926609 | C | A | -0.007435 | 0.001320 | 0.735838 | 2.50E-08 | 0.096261 | 0.088595 | 0.740965 | 2.77E-01 | TRUE | 9.06E-05 | 31.733 |
| Childhood sunburn occasions on vitiligo (main analysis) | | | | | | | | | | | | | |
| rs10168349 | C | G | 0.017342 | 0.002254 | 0.335704 | 1.40E-14 | -0.140949 | 0.086224 | 0.280817 | 1.02E-01 | TRUE | 1.71E-04 | 59.175 |
| rs10202908 | T | C | -0.012604 | 0.002271 | 0.674884 | 2.90E-08 | 0.076436 | 0.084188 | 0.699308 | 3.64E-01 | TRUE | 8.88E-05 | 30.797 |
| rs10220751 | G | T | -0.013690 | 0.002164 | 0.404814 | 2.50E-10 | 0.097347 | 0.080192 | 0.360712 | 2.25E-01 | TRUE | 1.15E-04 | 40.023 |
| rs10788627 | C | T | 0.012019 | 0.002129 | 0.474528 | 1.60E-08 | 0.194937 | 0.077296 | 0.545829 | 1.17E-02 | TRUE | 9.19E-05 | 31.873 |
| rs10810636 | G | A | 0.027509 | 0.002494 | 0.760343 | 2.80E-28 | 0.012809 | 0.087443 | 0.736142 | 8.84E-01 | TRUE | 3.50E-04 | 121.638 |
| rs10873552 | G | A | 0.012355 | 0.002244 | 0.657663 | 3.70E-08 | 0.028119 | 0.078192 | 0.572300 | 7.19E-01 | TRUE | 8.74E-05 | 30.323 |
| rs10896139 | T | C | -0.013634 | 0.002405 | 0.270905 | 1.40E-08 | -0.042024 | 0.086093 | 0.279817 | 6.25E-01 | TRUE | 9.26E-05 | 32.138 |
| rs11070811 | T | C | -0.017610 | 0.002749 | 0.183157 | 1.50E-10 | 0.192501 | 0.113156 | 0.133263 | 8.89E-02 | TRUE | 1.18E-04 | 41.048 |
| rs11104733 | T | C | 0.049182 | 0.008852 | 0.014717 | 2.80E-08 | -0.509677 | 0.254882 | 0.023720 | 4.55E-02 | TRUE | 8.90E-05 | 30.868 |
| rs111391498 | G | A | -0.047383 | 0.004975 | 0.047880 | 1.70E-21 | 0.408525 | 0.234693 | 0.028378 | 8.17E-02 | TRUE | 2.61E-04 | 90.720 |
| rs112089506 | T | C | -0.058086 | 0.004009 | 0.081196 | 1.40E-47 | 0.180376 | 0.134048 | 0.094132 | 1.78E-01 | TRUE | 6.05E-04 | 209.929 |
| rs11242899 | A | G | -0.024479 | 0.002411 | 0.266333 | 3.20E-24 | 0.094052 | 0.087927 | 0.261521 | 2.85E-01 | TRUE | 2.97E-04 | 103.074 |
| rs1126809 | A | G | 0.082500 | 0.002315 | 0.302839 | 1.00E-200 | -0.251597 | 0.100794 | 0.177232 | 1.26E-02 | TRUE | 3.65E-03 | 1270.330 |
| rs116125333 | G | T | -0.039708 | 0.007195 | 0.023181 | 3.40E-08 | 0.074232 | 0.140560 | 0.083237 | 5.97E-01 | TRUE | 8.78E-05 | 30.456 |
| rs11648436 | T | C | -0.027274 | 0.002219 | 0.356354 | 9.90E-35 | 0.011707 | 0.083466 | 0.307005 | 8.88E-01 | TRUE | 4.35E-04 | 151.104 |
| rs117132860 | A | G | 0.059305 | 0.006729 | 0.025574 | 1.20E-18 | -0.271036 | 0.606270 | 0.004001 | 6.55E-01 | TRUE | 2.24E-04 | 77.665 |
| rs11739906 | C | A | 0.013047 | 0.002265 | 0.327463 | 8.40E-09 | 0.086440 | 0.090270 | 0.240092 | 3.38E-01 | TRUE | 9.56E-05 | 33.189 |
| rs117462393 | T | C | 0.081482 | 0.009554 | 0.014374 | 1.50E-17 | 0.089137 | 0.156909 | 0.065008 | 5.70E-01 | TRUE | 2.10E-04 | 72.743 |
| rs12203592 | T | C | 0.153131 | 0.002526 | 0.219070 | 1.00E-200 | -0.155164 | 0.228257 | 0.030371 | 4.97E-01 | TRUE | 1.05E-02 | 3674.212 |
| rs1233578 | G | A | -0.020587 | 0.002787 | 0.176418 | 1.50E-13 | 0.040788 | 0.161190 | 0.060552 | 8.00E-01 | TRUE | 1.57E-04 | 54.565 |
| rs12350739 | A | G | 0.035464 | 0.002190 | 0.606787 | 5.80E-59 | 0.091953 | 0.077416 | 0.543926 | 2.35E-01 | TRUE | 7.55E-04 | 262.151 |
| rs1260326 | C | T | -0.012528 | 0.002173 | 0.603989 | 8.10E-09 | -0.112045 | 0.080859 | 0.650354 | 1.66E-01 | TRUE | 9.58E-05 | 33.254 |
| rs1267038 | C | A | -0.017889 | 0.002991 | 0.844933 | 2.20E-09 | -0.059347 | 0.100041 | 0.818008 | 5.53E-01 | TRUE | 1.03E-04 | 35.768 |
| rs1278766 | C | T | 0.019119 | 0.002139 | 0.545712 | 3.90E-19 | -0.019147 | 0.077117 | 0.486703 | 8.04E-01 | TRUE | 2.30E-04 | 79.905 |
| rs12913832 | G | A | 0.060538 | 0.002531 | 0.775275 | 1.90E-126 | 0.283293 | 0.129618 | 0.895544 | 2.88E-02 | TRUE | 1.65E-03 | 572.141 |
| rs1308048 | C | T | -0.020350 | 0.002164 | 0.419538 | 5.30E-21 | -0.164059 | 0.077709 | 0.429769 | 3.48E-02 | TRUE | 2.55E-04 | 88.406 |
| rs1326798 | G | C | 0.012077 | 0.002192 | 0.620628 | 3.60E-08 | -0.104696 | 0.080102 | 0.635754 | 1.91E-01 | TRUE | 8.75E-05 | 30.347 |
| rs13332673 | T | G | -0.102103 | 0.010535 | 0.010249 | 3.30E-22 | -0.008078 | 0.450346 | 0.007522 | 9.86E-01 | TRUE | 2.71E-04 | 93.934 |
| rs139414522 | C | T | -0.078388 | 0.009069 | 0.015298 | 5.40E-18 | -0.222158 | 0.400716 | 0.009396 | 5.79E-01 | TRUE | 2.15E-04 | 74.719 |
| rs141817469 | T | C | -0.093196 | 0.005820 | 0.035108 | 1.00E-57 | -0.110242 | 0.221606 | 0.031490 | 6.19E-01 | TRUE | 7.39E-04 | 256.425 |
| rs142314514 | G | A | -0.081593 | 0.007027 | 0.028510 | 3.60E-31 | -0.114638 | 0.250202 | 0.029766 | 6.47E-01 | TRUE | 3.88E-04 | 134.811 |
| rs1437635 | A | C | 0.020699 | 0.002867 | 0.166029 | 5.20E-13 | -0.035871 | 0.120568 | 0.115232 | 7.66E-01 | TRUE | 1.50E-04 | 52.127 |
| rs150527451 | A | G | 0.026240 | 0.003470 | 0.106417 | 4.00E-14 | -0.013814 | 0.091549 | 0.233292 | 8.80E-01 | TRUE | 1.65E-04 | 57.166 |
| rs151337382 | A | T | 0.057465 | 0.009250 | 0.013552 | 5.20E-10 | 0.214246 | 0.571593 | 0.004687 | 7.08E-01 | TRUE | 1.11E-04 | 38.594 |
| rs1548714 | C | A | 0.016158 | 0.002723 | 0.811733 | 3.00E-09 | -0.127519 | 0.099009 | 0.812556 | 1.98E-01 | TRUE | 1.01E-04 | 35.212 |
| rs16891982 | G | C | 0.229157 | 0.006209 | 0.970879 | 1.00E-200 | -0.105041 | 0.305363 | 0.982617 | 7.31E-01 | TRUE | 3.91E-03 | 1362.250 |
| rs17232484 | A | G | 0.061024 | 0.009074 | 0.014194 | 1.80E-11 | 0.212095 | 0.290802 | 0.018867 | 4.66E-01 | TRUE | 1.30E-04 | 45.227 |
| rs1805007 | T | C | 0.196373 | 0.003503 | 0.100947 | 1.00E-200 | -0.224255 | 0.156542 | 0.066054 | 1.52E-01 | TRUE | 8.98E-03 | 3142.324 |
| rs1805008 | T | C | 0.119469 | 0.003767 | 0.086593 | 1.00E-200 | -0.193515 | 0.152876 | 0.068151 | 2.06E-01 | TRUE | 2.89E-03 | 1005.614 |
| rs1989483 | G | A | 0.012496 | 0.002190 | 0.389054 | 1.20E-08 | -0.060832 | 0.079833 | 0.369753 | 4.46E-01 | TRUE | 9.39E-05 | 32.568 |
| rs2299098 | C | G | 0.014791 | 0.002684 | 0.196234 | 3.60E-08 | 0.131784 | 0.106299 | 0.154392 | 2.15E-01 | TRUE | 8.76E-05 | 30.379 |
| rs251468 | T | C | -0.028198 | 0.002474 | 0.248353 | 4.40E-30 | 0.155735 | 0.081170 | 0.345127 | 5.50E-02 | TRUE | 3.74E-04 | 129.875 |
| rs2737217 | G | A | -0.025456 | 0.002158 | 0.563309 | 4.10E-32 | 0.102291 | 0.082192 | 0.665199 | 2.13E-01 | TRUE | 4.01E-04 | 139.161 |
| rs3213737 | A | G | -0.031866 | 0.002159 | 0.576000 | 2.70E-49 | 0.044151 | 0.081081 | 0.655436 | 5.86E-01 | TRUE | 6.27E-04 | 217.793 |
| rs35563099 | T | C | -0.031031 | 0.002915 | 0.164009 | 1.80E-26 | 0.001831 | 0.100039 | 0.184561 | 9.85E-01 | TRUE | 3.27E-04 | 113.328 |
| rs3759579 | G | A | 0.012449 | 0.002159 | 0.588839 | 8.10E-09 | -0.023253 | 0.079307 | 0.619894 | 7.69E-01 | TRUE | 9.58E-05 | 33.241 |
| rs41563 | A | G | 0.013506 | 0.002232 | 0.349781 | 1.40E-09 | 0.039810 | 0.083750 | 0.306902 | 6.35E-01 | TRUE | 1.05E-04 | 36.607 |
| rs4240559 | C | T | -0.017105 | 0.002143 | 0.557906 | 1.40E-15 | 0.115587 | 0.080707 | 0.652173 | 1.52E-01 | TRUE | 1.84E-04 | 63.732 |
| rs4272574 | T | C | 0.015232 | 0.002129 | 0.478288 | 8.40E-13 | -0.014132 | 0.077167 | 0.509256 | 8.55E-01 | TRUE | 1.48E-04 | 51.189 |
| rs4335021 | C | T | -0.011791 | 0.002162 | 0.599720 | 4.90E-08 | 0.281901 | 0.079255 | 0.639191 | 3.75E-04 | TRUE | 8.58E-05 | 29.755 |
| rs4438032 | G | C | 0.023197 | 0.003647 | 0.906248 | 2.00E-10 | -0.177765 | 0.150350 | 0.927912 | 2.37E-01 | TRUE | 1.17E-04 | 40.465 |
| rs4578351 | C | T | -0.020481 | 0.002572 | 0.222048 | 1.70E-15 | 0.071568 | 0.089639 | 0.247812 | 4.25E-01 | TRUE | 1.83E-04 | 63.395 |
| rs4670813 | A | G | -0.012669 | 0.002144 | 0.470775 | 3.50E-09 | 0.069095 | 0.077370 | 0.524672 | 3.72E-01 | TRUE | 1.01E-04 | 34.910 |
| rs4840542 | T | G | 0.018509 | 0.002133 | 0.505804 | 4.00E-18 | -0.153497 | 0.078345 | 0.426420 | 5.01E-02 | TRUE | 2.17E-04 | 75.304 |
| rs511515 | G | A | -0.020386 | 0.002321 | 0.700890 | 1.60E-18 | 0.207585 | 0.092914 | 0.781183 | 2.55E-02 | TRUE | 2.22E-04 | 77.130 |
| rs537894 | A | G | 0.012726 | 0.002177 | 0.548976 | 5.00E-09 | 0.154387 | 0.082663 | 0.671718 | 6.18E-02 | TRUE | 9.85E-05 | 34.171 |
| rs57994353 | C | T | 0.012726 | 0.002323 | 0.299074 | 4.30E-08 | -0.015012 | 0.088534 | 0.254535 | 8.65E-01 | TRUE | 8.65E-05 | 30.017 |
| rs6007506 | T | C | -0.024813 | 0.002257 | 0.337443 | 4.10E-28 | -0.103816 | 0.080213 | 0.356734 | 1.96E-01 | TRUE | 3.48E-04 | 120.838 |
| rs6059655 | G | A | -0.127339 | 0.003580 | 0.897978 | 1.00E-200 | -0.227909 | 0.258239 | 0.977546 | 3.77E-01 | TRUE | 3.63E-03 | 1265.313 |
| rs61816766 | C | T | 0.036578 | 0.006188 | 0.032281 | 3.40E-09 | 0.340348 | 0.425563 | 0.007738 | 4.24E-01 | TRUE | 1.01E-04 | 34.942 |
| rs61981034 | A | G | 0.013326 | 0.002438 | 0.257954 | 4.60E-08 | -0.086881 | 0.081623 | 0.336588 | 2.87E-01 | TRUE | 8.61E-05 | 29.878 |
| rs6689641 | G | A | 0.014934 | 0.002134 | 0.542588 | 2.60E-12 | -0.032929 | 0.077671 | 0.560178 | 6.72E-01 | TRUE | 1.41E-04 | 48.962 |
| rs6882046 | G | A | 0.014350 | 0.002422 | 0.267930 | 3.10E-09 | -0.035006 | 0.100295 | 0.180398 | 7.27E-01 | TRUE | 1.01E-04 | 35.103 |
| rs699780 | G | A | -0.018449 | 0.003185 | 0.127758 | 7.00E-09 | 0.089148 | 0.102867 | 0.168039 | 3.86E-01 | TRUE | 9.67E-05 | 33.542 |
| rs72821630 | T | C | -0.015041 | 0.002455 | 0.251407 | 9.00E-10 | 0.150737 | 0.079947 | 0.363876 | 5.94E-02 | TRUE | 1.08E-04 | 37.527 |
| rs75300484 | T | C | -0.034208 | 0.005802 | 0.034690 | 3.70E-09 | 0.053386 | 0.254005 | 0.023242 | 8.34E-01 | TRUE | 1.00E-04 | 34.763 |
| rs7768317 | T | C | -0.017032 | 0.002465 | 0.248878 | 4.90E-12 | -0.113762 | 0.087785 | 0.261317 | 1.95E-01 | TRUE | 1.38E-04 | 47.730 |
| rs784235 | G | A | -0.016385 | 0.002763 | 0.819090 | 3.00E-09 | 0.087737 | 0.109759 | 0.855379 | 4.24E-01 | TRUE | 1.01E-04 | 35.179 |
| rs78444298 | A | G | -0.046672 | 0.007712 | 0.019692 | 1.40E-09 | 0.327071 | 0.294340 | 0.017698 | 2.66E-01 | TRUE | 1.06E-04 | 36.629 |
| rs849138 | A | G | 0.012559 | 0.002131 | 0.507025 | 3.80E-09 | 0.010458 | 0.077279 | 0.492330 | 8.92E-01 | TRUE | 1.00E-04 | 34.732 |
| rs9328259 | A | C | 0.026744 | 0.002370 | 0.718185 | 1.60E-29 | -0.083084 | 0.086127 | 0.721066 | 3.35E-01 | TRUE | 3.67E-04 | 127.323 |
| rs9821675 | G | A | -0.012037 | 0.002125 | 0.505270 | 1.50E-08 | -0.015918 | 0.078726 | 0.395061 | 8.40E-01 | TRUE | 9.25E-05 | 32.087 |
| rs9832130 | A | G | -0.012794 | 0.002162 | 0.580680 | 3.30E-09 | -0.060552 | 0.077748 | 0.563684 | 4.36E-01 | TRUE | 1.01E-04 | 35.010 |
| rs9835772 | T | A | 0.017716 | 0.002477 | 0.243745 | 8.60E-13 | 0.054960 | 0.091535 | 0.229146 | 5.48E-01 | TRUE | 1.47E-04 | 51.137 |
| rs9858244 | A | G | 0.018945 | 0.002597 | 0.214182 | 3.00E-13 | 0.055900 | 0.099321 | 0.184634 | 5.74E-01 | TRUE | 1.53E-04 | 53.225 |
| rs9867857 | T | C | 0.013625 | 0.002136 | 0.489160 | 1.80E-10 | -0.082194 | 0.077943 | 0.429514 | 2.92E-01 | TRUE | 1.17E-04 | 40.701 |
| Ease of skin tanning on vitiligo (main analysis) | | | | | | | | | | | | | |
| rs10054039 | A | G | -0.012765 | 0.002077 | 0.373657 | 8.00E-10 | 0.001422 | 0.078377 | 0.422053 | 9.86E-01 | TRUE | 8.34E-05 | 37.771 |
| rs10202360 | C | A | -0.021801 | 0.002627 | 0.177348 | 1.00E-16 | 0.057544 | 0.088602 | 0.256664 | 5.16E-01 | TRUE | 1.52E-04 | 68.881 |
| rs10273864 | C | A | -0.011537 | 0.002019 | 0.536443 | 1.10E-08 | 0.043881 | 0.077364 | 0.520230 | 5.71E-01 | TRUE | 7.21E-05 | 32.650 |
| rs1031896 | C | T | -0.011944 | 0.002077 | 0.382321 | 8.90E-09 | 0.045938 | 0.080632 | 0.353093 | 5.69E-01 | TRUE | 7.30E-05 | 33.075 |
| rs10794666 | T | C | 0.015740 | 0.002024 | 0.572003 | 7.40E-15 | 0.033592 | 0.077216 | 0.550094 | 6.64E-01 | TRUE | 1.34E-04 | 60.495 |
| rs10810636 | G | A | 0.056176 | 0.002351 | 0.760701 | 3.70E-126 | 0.012809 | 0.087443 | 0.736142 | 8.84E-01 | TRUE | 1.26E-03 | 570.806 |
| rs10859995 | C | T | 0.011452 | 0.002035 | 0.581427 | 1.80E-08 | 0.043457 | 0.081558 | 0.662999 | 5.94E-01 | TRUE | 6.99E-05 | 31.672 |
| rs10879095 | A | G | -0.013180 | 0.002087 | 0.639015 | 2.70E-10 | -0.196419 | 0.084040 | 0.697553 | 1.94E-02 | TRUE | 8.80E-05 | 39.891 |
| rs10897156 | A | T | 0.095083 | 0.012068 | 0.993027 | 3.30E-15 | 0.518381 | 0.363962 | 0.988110 | 1.54E-01 | TRUE | 1.37E-04 | 62.075 |
| rs10947996 | T | G | -0.018736 | 0.002305 | 0.251254 | 4.30E-16 | -0.211429 | 0.087289 | 0.266378 | 1.54E-02 | TRUE | 1.46E-04 | 66.075 |
| rs11070811 | T | C | -0.029807 | 0.002589 | 0.182822 | 1.10E-30 | 0.192501 | 0.113156 | 0.133263 | 8.89E-02 | TRUE | 2.93E-04 | 132.562 |
| rs11104947 | A | G | 0.118269 | 0.008225 | 0.015194 | 7.10E-47 | -0.402363 | 0.249383 | 0.024863 | 1.07E-01 | TRUE | 4.56E-04 | 206.738 |
| rs111310469 | T | C | 0.045452 | 0.007613 | 0.018410 | 2.40E-09 | -0.709940 | 0.473905 | 0.007015 | 1.34E-01 | TRUE | 7.87E-05 | 35.648 |
| rs112089506 | T | C | -0.137978 | 0.003735 | 0.080846 | 1.00E-200 | 0.180376 | 0.134048 | 0.094132 | 1.78E-01 | TRUE | 3.00E-03 | 1364.954 |
| rs112217744 | A | G | -0.035499 | 0.004893 | 0.043784 | 4.00E-13 | 0.030409 | 0.145268 | 0.075616 | 8.34E-01 | TRUE | 1.16E-04 | 52.640 |
| rs1123483 | G | A | 0.015598 | 0.002110 | 0.657258 | 1.50E-13 | -0.076625 | 0.084040 | 0.701368 | 3.62E-01 | TRUE | 1.21E-04 | 54.622 |
| rs11242899 | A | G | -0.033873 | 0.002269 | 0.266624 | 2.10E-50 | 0.094052 | 0.087927 | 0.261521 | 2.85E-01 | TRUE | 4.92E-04 | 222.939 |
| rs1126809 | A | G | 0.129574 | 0.002176 | 0.304042 | 1.00E-200 | -0.251597 | 0.100794 | 0.177232 | 1.26E-02 | TRUE | 7.77E-03 | 3546.271 |
| rs1129614 | A | G | -0.016602 | 0.002445 | 0.214257 | 1.10E-11 | -0.106256 | 0.094492 | 0.209501 | 2.61E-01 | TRUE | 1.02E-04 | 46.126 |
| rs113151897 | T | A | -0.021676 | 0.003721 | 0.080142 | 5.70E-09 | -0.276081 | 0.120310 | 0.118110 | 2.17E-02 | TRUE | 7.49E-05 | 33.939 |
| rs113287072 | C | T | -0.200779 | 0.009410 | 0.011960 | 5.30E-101 | 0.395536 | 0.483076 | 0.007503 | 4.13E-01 | TRUE | 1.00E-03 | 455.213 |
| rs113455175 | T | C | 0.020715 | 0.003044 | 0.124301 | 1.00E-11 | 0.131702 | 0.105016 | 0.159085 | 2.10E-01 | TRUE | 1.02E-04 | 46.304 |
| rs1137134 | A | G | 0.043611 | 0.002047 | 0.602148 | 1.10E-100 | -0.115276 | 0.079518 | 0.624022 | 1.47E-01 | TRUE | 1.00E-03 | 453.679 |
| rs11639071 | G | A | 0.012071 | 0.002035 | 0.431625 | 3.00E-09 | 0.018022 | 0.078558 | 0.397816 | 8.19E-01 | TRUE | 7.77E-05 | 35.196 |
| rs117132860 | A | G | 0.148572 | 0.006304 | 0.025972 | 7.91E-123 | -0.271036 | 0.606270 | 0.004001 | 6.55E-01 | TRUE | 1.22E-03 | 555.514 |
| rs11907438 | A | T | -0.052101 | 0.003620 | 0.083291 | 5.70E-47 | 0.226697 | 0.114387 | 0.132050 | 4.75E-02 | TRUE | 4.57E-04 | 207.162 |
| rs11954036 | C | T | 0.023666 | 0.002138 | 0.326762 | 1.80E-28 | 0.087724 | 0.090301 | 0.240456 | 3.31E-01 | TRUE | 2.70E-04 | 122.530 |
| rs12146565 | A | G | 0.013887 | 0.002142 | 0.321425 | 9.00E-11 | -0.069703 | 0.083036 | 0.313320 | 4.01E-01 | TRUE | 9.28E-05 | 42.029 |
| rs12203592 | T | C | 0.261262 | 0.002376 | 0.219829 | 1.00E-200 | -0.155164 | 0.228257 | 0.030371 | 4.97E-01 | TRUE | 2.60E-02 | 12085.991 |
| rs12350739 | A | G | 0.074071 | 0.002066 | 0.607970 | 1.00E-200 | 0.091953 | 0.077416 | 0.543926 | 2.35E-01 | TRUE | 2.83E-03 | 1285.261 |
| rs12550943 | A | G | 0.012409 | 0.002221 | 0.289927 | 2.30E-08 | 0.000763 | 0.081389 | 0.337618 | 9.93E-01 | TRUE | 6.89E-05 | 31.229 |
| rs12896063 | A | G | 0.011120 | 0.002024 | 0.564339 | 3.90E-08 | -0.059213 | 0.079048 | 0.615025 | 4.54E-01 | TRUE | 6.66E-05 | 30.176 |
| rs12913832 | G | A | 0.154261 | 0.002390 | 0.777053 | 1.00E-200 | 0.283293 | 0.129618 | 0.895544 | 2.88E-02 | TRUE | 9.11E-03 | 4164.456 |
| rs1295621 | A | T | 0.013374 | 0.002392 | 0.226125 | 2.20E-08 | 0.033533 | 0.093061 | 0.218249 | 7.19E-01 | TRUE | 6.90E-05 | 31.269 |
| rs137879272 | A | C | -0.201986 | 0.008973 | 0.014126 | 3.30E-112 | -0.055099 | 0.395645 | 0.009973 | 8.89E-01 | TRUE | 1.12E-03 | 506.701 |
| rs141210533 | A | G | -0.122844 | 0.010204 | 0.011050 | 2.20E-33 | 0.256144 | 0.199466 | 0.039216 | 1.99E-01 | TRUE | 3.20E-04 | 144.927 |
| rs141817469 | T | C | -0.177358 | 0.005426 | 0.034919 | 1.00E-200 | -0.110242 | 0.221606 | 0.031490 | 6.19E-01 | TRUE | 2.35E-03 | 1068.262 |
| rs142314514 | G | A | -0.185300 | 0.006557 | 0.028340 | 1.10E-175 | -0.114638 | 0.250202 | 0.029766 | 6.47E-01 | TRUE | 1.76E-03 | 798.534 |
| rs146048597 | A | G | -0.017282 | 0.003002 | 0.123531 | 8.60E-09 | -0.005508 | 0.105351 | 0.160293 | 9.58E-01 | TRUE | 7.31E-05 | 33.131 |
| rs147535976 | A | G | -0.100712 | 0.012236 | 0.006870 | 1.90E-16 | -1.130660 | 2.235860 | 0.000368 | 6.13E-01 | TRUE | 1.50E-04 | 67.743 |
| rs1483734 | C | T | -0.013418 | 0.002383 | 0.233494 | 1.80E-08 | -0.187016 | 0.105090 | 0.161809 | 7.51E-02 | TRUE | 7.00E-05 | 31.705 |
| rs1493698 | C | T | -0.018357 | 0.003043 | 0.124181 | 1.60E-09 | 0.091296 | 0.102920 | 0.167731 | 3.75E-01 | TRUE | 8.03E-05 | 36.401 |
| rs1613999 | G | T | -0.034218 | 0.002039 | 0.421009 | 3.50E-63 | -0.160452 | 0.077719 | 0.433665 | 3.90E-02 | TRUE | 6.21E-04 | 281.525 |
| rs16891982 | G | C | 0.406336 | 0.006001 | 0.972206 | 1.00E-200 | -0.105041 | 0.305363 | 0.982617 | 7.31E-01 | TRUE | 1.00E-02 | 4584.276 |
| rs17232484 | A | G | 0.123729 | 0.008414 | 0.014260 | 6.00E-49 | 0.212095 | 0.290802 | 0.018867 | 4.66E-01 | TRUE | 4.77E-04 | 216.245 |
| rs17458491 | A | C | 0.011257 | 0.002026 | 0.561825 | 2.70E-08 | 0.057573 | 0.076904 | 0.525452 | 4.54E-01 | TRUE | 6.82E-05 | 30.880 |
| rs177114 | C | A | 0.011353 | 0.002008 | 0.524990 | 1.60E-08 | 0.001003 | 0.077093 | 0.536839 | 9.90E-01 | TRUE | 7.06E-05 | 31.969 |
| rs1800440 | C | T | -0.034060 | 0.002573 | 0.186036 | 5.40E-40 | 0.090386 | 0.108501 | 0.148598 | 4.05E-01 | TRUE | 3.87E-04 | 175.196 |
| rs1805007 | T | C | 0.427724 | 0.003233 | 0.102727 | 1.00E-200 | -0.224255 | 0.156542 | 0.066054 | 1.52E-01 | TRUE | 3.72E-02 | 17499.593 |
| rs1805008 | T | C | 0.305776 | 0.003486 | 0.087531 | 1.00E-200 | -0.193515 | 0.152876 | 0.068151 | 2.06E-01 | TRUE | 1.67E-02 | 7694.846 |
| rs1870940 | A | G | 0.020411 | 0.002271 | 0.271365 | 2.50E-19 | -0.033822 | 0.090609 | 0.236294 | 7.09E-01 | TRUE | 1.78E-04 | 80.815 |
| rs1885194 | C | T | 0.035229 | 0.002017 | 0.448275 | 2.70E-68 | 0.101912 | 0.077052 | 0.470212 | 1.86E-01 | TRUE | 6.73E-04 | 305.019 |
| rs2094756 | C | A | -0.067579 | 0.010845 | 0.009003 | 4.60E-10 | 0.077313 | 0.281748 | 0.019461 | 7.84E-01 | TRUE | 8.57E-05 | 38.833 |
| rs2167957 | T | C | 0.017282 | 0.002311 | 0.745779 | 7.60E-14 | -0.056062 | 0.086317 | 0.722297 | 5.16E-01 | TRUE | 1.23E-04 | 55.900 |
| rs2236295 | T | G | -0.015876 | 0.002053 | 0.403189 | 1.10E-14 | 0.100682 | 0.080128 | 0.366336 | 2.09E-01 | TRUE | 1.32E-04 | 59.771 |
| rs251466 | G | C | -0.036884 | 0.002330 | 0.248491 | 1.90E-56 | 0.156125 | 0.081159 | 0.345025 | 5.44E-02 | TRUE | 5.53E-04 | 250.624 |
| rs2737207 | A | C | -0.044273 | 0.002027 | 0.556779 | 8.79E-106 | 0.120865 | 0.083670 | 0.688067 | 1.49E-01 | TRUE | 1.05E-03 | 477.166 |
| rs308896 | T | G | -0.075160 | 0.007506 | 0.018457 | 1.30E-23 | 0.011126 | 0.193753 | 0.041120 | 9.54E-01 | TRUE | 2.21E-04 | 100.266 |
| rs329120 | T | C | 0.011380 | 0.002034 | 0.419224 | 2.20E-08 | -0.073829 | 0.077933 | 0.424469 | 3.43E-01 | TRUE | 6.91E-05 | 31.289 |
| rs34635363 | A | G | 0.023500 | 0.002093 | 0.358436 | 3.00E-29 | 0.008315 | 0.079363 | 0.386424 | 9.17E-01 | TRUE | 2.78E-04 | 126.061 |
| rs35380972 | C | A | -0.020656 | 0.002083 | 0.368172 | 3.50E-23 | 0.060558 | 0.085778 | 0.280590 | 4.80E-01 | TRUE | 2.17E-04 | 98.345 |
| rs3743538 | T | G | -0.016591 | 0.002065 | 0.352448 | 9.40E-16 | -0.017066 | 0.083782 | 0.302835 | 8.39E-01 | TRUE | 1.42E-04 | 64.562 |
| rs3851294 | G | A | -0.047498 | 0.003456 | 0.907723 | 5.70E-43 | -0.035760 | 0.140614 | 0.918521 | 7.99E-01 | TRUE | 4.17E-04 | 188.853 |
| rs399856 | A | G | -0.015868 | 0.002074 | 0.620560 | 2.00E-14 | 0.068284 | 0.077274 | 0.518142 | 3.77E-01 | TRUE | 1.29E-04 | 58.507 |
| rs4257287 | T | C | -0.018601 | 0.003199 | 0.887895 | 6.00E-09 | 0.025618 | 0.111161 | 0.861112 | 8.18E-01 | TRUE | 7.46E-05 | 33.819 |
| rs4406278 | T | G | 0.042633 | 0.002229 | 0.718260 | 1.50E-81 | -0.082811 | 0.086130 | 0.721074 | 3.36E-01 | TRUE | 8.07E-04 | 365.898 |
| rs4499192 | G | A | 0.163989 | 0.011934 | 0.991314 | 5.80E-43 | -0.095994 | 0.230793 | 0.970717 | 6.77E-01 | TRUE | 4.17E-04 | 188.811 |
| rs4592422 | T | C | 0.066607 | 0.003434 | 0.906242 | 8.00E-84 | -0.162737 | 0.150623 | 0.928388 | 2.80E-01 | TRUE | 8.30E-04 | 376.295 |
| rs4689314 | C | T | 0.023449 | 0.004077 | 0.935179 | 8.90E-09 | -0.010569 | 0.234616 | 0.972336 | 9.64E-01 | TRUE | 7.30E-05 | 33.073 |
| rs4756838 | T | C | 0.016558 | 0.002698 | 0.167791 | 8.40E-10 | 0.009301 | 0.118048 | 0.121441 | 9.37E-01 | TRUE | 8.31E-05 | 37.656 |
| rs4760521 | A | G | 0.013474 | 0.002091 | 0.367303 | 1.20E-10 | 0.130092 | 0.079276 | 0.380906 | 1.01E-01 | TRUE | 9.17E-05 | 41.536 |
| rs4778984 | G | A | 0.015267 | 0.002289 | 0.256877 | 2.60E-11 | -0.037269 | 0.099105 | 0.183939 | 7.07E-01 | TRUE | 9.82E-05 | 44.476 |
| rs4788566 | C | T | 0.018817 | 0.002230 | 0.732878 | 3.20E-17 | 0.047264 | 0.092720 | 0.778097 | 6.10E-01 | TRUE | 1.57E-04 | 71.217 |
| rs487490 | T | C | -0.022552 | 0.003989 | 0.067522 | 1.60E-08 | -0.187770 | 0.176460 | 0.049490 | 2.87E-01 | TRUE | 7.06E-05 | 31.970 |
| rs4933379 | T | C | 0.012754 | 0.002015 | 0.468545 | 2.40E-10 | 0.123635 | 0.077162 | 0.523849 | 1.09E-01 | TRUE | 8.85E-05 | 40.081 |
| rs4939832 | G | A | -0.013217 | 0.002354 | 0.242885 | 2.00E-08 | -0.047472 | 0.088728 | 0.253874 | 5.93E-01 | TRUE | 6.96E-05 | 31.538 |
| rs6007506 | T | C | -0.036322 | 0.002131 | 0.336835 | 3.90E-65 | -0.103816 | 0.080213 | 0.356734 | 1.96E-01 | TRUE | 6.41E-04 | 290.503 |
| rs60143966 | A | C | -0.015854 | 0.002379 | 0.239145 | 2.60E-11 | 0.027285 | 0.090648 | 0.240513 | 7.63E-01 | TRUE | 9.80E-05 | 44.426 |
| rs6059013 | A | G | -0.016798 | 0.002343 | 0.240526 | 7.50E-13 | -0.101295 | 0.085715 | 0.283559 | 2.37E-01 | TRUE | 1.13E-04 | 51.415 |
| rs6059655 | G | A | -0.273292 | 0.003350 | 0.896917 | 1.00E-200 | -0.227909 | 0.258239 | 0.977546 | 3.77E-01 | TRUE | 1.45E-02 | 6653.629 |
| rs6061182 | C | T | -0.011362 | 0.002017 | 0.562875 | 1.80E-08 | -0.048170 | 0.080306 | 0.646113 | 5.49E-01 | TRUE | 7.01E-05 | 31.742 |
| rs60780889 | T | C | -0.020057 | 0.002286 | 0.259227 | 1.70E-18 | 0.032758 | 0.089291 | 0.245926 | 7.14E-01 | TRUE | 1.70E-04 | 76.959 |
| rs6088232 | T | C | -0.062364 | 0.007842 | 0.017951 | 1.80E-15 | 0.004735 | 0.175687 | 0.050375 | 9.78E-01 | TRUE | 1.40E-04 | 63.244 |
| rs61348812 | C | T | 0.014761 | 0.002570 | 0.811921 | 9.20E-09 | -0.127155 | 0.098994 | 0.812544 | 1.99E-01 | TRUE | 7.28E-05 | 32.999 |
| rs61935849 | C | A | 0.027523 | 0.003185 | 0.111948 | 5.50E-18 | 0.290394 | 0.141821 | 0.079858 | 4.06E-02 | TRUE | 1.65E-04 | 74.691 |
| rs62435865 | C | T | 0.021432 | 0.003162 | 0.115279 | 1.20E-11 | -0.194053 | 0.117256 | 0.123219 | 9.79E-02 | TRUE | 1.01E-04 | 45.945 |
| rs6681924 | G | A | -0.013053 | 0.002316 | 0.750657 | 1.70E-08 | 0.131447 | 0.086968 | 0.731912 | 1.31E-01 | TRUE | 7.01E-05 | 31.756 |
| rs6689641 | G | A | 0.016560 | 0.002011 | 0.542975 | 1.80E-16 | -0.032929 | 0.077671 | 0.560178 | 6.72E-01 | TRUE | 1.50E-04 | 67.807 |
| rs670318 | C | T | -0.046988 | 0.004666 | 0.951699 | 7.60E-24 | -0.070260 | 0.162159 | 0.939656 | 6.65E-01 | TRUE | 2.24E-04 | 101.391 |
| rs6790699 | G | A | 0.019593 | 0.002072 | 0.626661 | 3.30E-21 | -0.026195 | 0.085623 | 0.716909 | 7.60E-01 | TRUE | 1.97E-04 | 89.372 |
| rs68099344 | T | A | 0.018704 | 0.002645 | 0.174310 | 1.50E-12 | 0.089575 | 0.081458 | 0.341342 | 2.71E-01 | TRUE | 1.10E-04 | 50.021 |
| rs68173807 | G | A | 0.012882 | 0.002137 | 0.329079 | 1.70E-09 | 0.079674 | 0.082294 | 0.330092 | 3.33E-01 | TRUE | 8.02E-05 | 36.328 |
| rs6879874 | T | A | -0.015389 | 0.002243 | 0.723617 | 6.80E-12 | -0.085788 | 0.083569 | 0.696455 | 3.05E-01 | TRUE | 1.04E-04 | 47.083 |
| rs6914598 | C | T | 0.014839 | 0.002153 | 0.323950 | 5.40E-12 | -0.008359 | 0.082721 | 0.318104 | 9.20E-01 | TRUE | 1.05E-04 | 47.523 |
| rs6966404 | G | T | 0.021362 | 0.003577 | 0.086710 | 2.40E-09 | 0.025916 | 0.163837 | 0.058939 | 8.74E-01 | TRUE | 7.87E-05 | 35.658 |
| rs7098111 | T | C | -0.056163 | 0.002756 | 0.163162 | 2.50E-92 | 0.003089 | 0.100207 | 0.184436 | 9.75E-01 | TRUE | 9.16E-04 | 415.326 |
| rs7246261 | T | C | 0.026242 | 0.004469 | 0.053352 | 4.30E-09 | -0.391792 | 0.163673 | 0.058185 | 1.67E-02 | TRUE | 7.61E-05 | 34.486 |
| rs7249850 | A | G | -0.011162 | 0.002032 | 0.428022 | 4.00E-08 | 0.202044 | 0.081247 | 0.345546 | 1.29E-02 | TRUE | 6.66E-05 | 30.158 |
| rs72632979 | G | A | -0.022156 | 0.002660 | 0.172279 | 8.10E-17 | 0.026255 | 0.151118 | 0.069820 | 8.62E-01 | TRUE | 1.53E-04 | 69.378 |
| rs726558 | G | A | -0.012957 | 0.002260 | 0.298149 | 9.90E-09 | -0.186002 | 0.096053 | 0.202909 | 5.28E-02 | TRUE | 7.25E-05 | 32.857 |
| rs72708989 | T | C | 0.037140 | 0.005103 | 0.040109 | 3.40E-13 | 0.117090 | 0.191562 | 0.042238 | 5.41E-01 | TRUE | 1.17E-04 | 52.969 |
| rs72710504 | T | C | 0.056578 | 0.007651 | 0.017589 | 1.40E-13 | 0.174808 | 0.446072 | 0.007251 | 6.95E-01 | TRUE | 1.21E-04 | 54.688 |
| rs72917317 | G | T | 0.085858 | 0.003253 | 0.107146 | 1.70E-153 | -0.020610 | 0.090691 | 0.239666 | 8.20E-01 | TRUE | 1.53E-03 | 696.478 |
| rs72954723 | A | G | -0.040519 | 0.006528 | 0.024086 | 5.40E-10 | -0.727304 | 0.380671 | 0.010429 | 5.61E-02 | TRUE | 8.50E-05 | 38.524 |
| rs7300019 | C | A | 0.103359 | 0.007733 | 0.020379 | 9.60E-41 | -0.435616 | 0.256463 | 0.023525 | 8.94E-02 | TRUE | 3.94E-04 | 178.637 |
| rs73087086 | G | A | 0.015145 | 0.002392 | 0.227840 | 2.40E-10 | -0.203453 | 0.107518 | 0.151836 | 5.85E-02 | TRUE | 8.85E-05 | 40.079 |
| rs75223371 | A | G | -0.034140 | 0.004856 | 0.044727 | 2.00E-12 | -0.008761 | 0.148617 | 0.071779 | 9.53E-01 | TRUE | 1.09E-04 | 49.435 |
| rs75300484 | T | C | -0.055748 | 0.005403 | 0.034584 | 5.90E-25 | 0.053386 | 0.254005 | 0.023242 | 8.34E-01 | TRUE | 2.35E-04 | 106.455 |
| rs75555084 | G | T | 0.086385 | 0.010820 | 0.008667 | 1.40E-15 | -0.169902 | 0.153280 | 0.068533 | 2.68E-01 | TRUE | 1.41E-04 | 63.742 |
| rs75575928 | T | C | 0.014042 | 0.002389 | 0.232603 | 4.10E-09 | -0.017391 | 0.086690 | 0.272979 | 8.41E-01 | TRUE | 7.63E-05 | 34.563 |
| rs7589217 | G | A | -0.011250 | 0.002047 | 0.399191 | 3.90E-08 | 0.012679 | 0.078138 | 0.427256 | 8.71E-01 | TRUE | 6.67E-05 | 30.202 |
| rs77568325 | G | A | -0.040219 | 0.006431 | 0.025035 | 4.00E-10 | 0.083329 | 0.209132 | 0.035798 | 6.90E-01 | TRUE | 8.63E-05 | 39.114 |
| rs77580397 | T | C | 0.013791 | 0.002486 | 0.205120 | 2.90E-08 | 0.144385 | 0.099051 | 0.186231 | 1.45E-01 | TRUE | 6.79E-05 | 30.766 |
| rs8007832 | C | T | 0.011813 | 0.002098 | 0.363861 | 1.80E-08 | 0.002967 | 0.080046 | 0.367921 | 9.70E-01 | TRUE | 6.99E-05 | 31.691 |
| rs8023038 | A | G | 0.011990 | 0.002135 | 0.656699 | 2.00E-08 | 0.001055 | 0.078351 | 0.570392 | 9.89E-01 | TRUE | 6.96E-05 | 31.544 |
| rs8028550 | C | T | 0.012368 | 0.002048 | 0.599338 | 1.50E-09 | -0.117738 | 0.077369 | 0.537184 | 1.28E-01 | TRUE | 8.05E-05 | 36.481 |
| rs8182578 | A | G | 0.011260 | 0.002039 | 0.430283 | 3.40E-08 | 0.034502 | 0.077993 | 0.429881 | 6.58E-01 | TRUE | 6.73E-05 | 30.493 |
| rs849138 | A | G | 0.017948 | 0.002009 | 0.507044 | 4.10E-19 | 0.010458 | 0.077279 | 0.492330 | 8.92E-01 | TRUE | 1.76E-04 | 79.824 |
| rs899381 | C | T | -0.070663 | 0.009689 | 0.010992 | 3.00E-13 | -0.070488 | 0.267199 | 0.021722 | 7.92E-01 | TRUE | 1.17E-04 | 53.190 |
| rs9302151 | C | T | -0.011835 | 0.002003 | 0.528038 | 3.40E-09 | -0.102579 | 0.077402 | 0.556616 | 1.85E-01 | TRUE | 7.71E-05 | 34.915 |
| rs9561570 | T | G | 0.021589 | 0.002163 | 0.314884 | 1.80E-23 | 0.015753 | 0.080383 | 0.371084 | 8.45E-01 | TRUE | 2.20E-04 | 99.659 |
| rs9821965 | G | A | -0.012202 | 0.002050 | 0.396818 | 2.60E-09 | 0.057049 | 0.077755 | 0.426119 | 4.63E-01 | TRUE | 7.82E-05 | 35.441 |
| rs9867857 | T | C | 0.023701 | 0.002015 | 0.489741 | 6.00E-32 | -0.082194 | 0.077943 | 0.429514 | 2.92E-01 | TRUE | 3.05E-04 | 138.387 |

**Table S5.** Instrumental variables used in validation analysis.

| SNP | Effect allele | Other  allele | beta.exposure | se.exposure | eaf.exposure | pval.exposure | beta.outcome | se.outcome | eaf.outcome | pval.outcome | Steiger direction | R^2^ | F |
| --- | --- | --- | --- | --- | --- | --- | --- | --- | --- | --- | --- | --- | --- |
| Sunburns on vitiligo (validation analysis) | | | | | | | | | | | | | |
| rs10168349 | C | G | 0.008641 | 0.00123311 | 0.335454 | 1.20E-13 | 0.000000 | 0.03 | 0.33 | 9.39E-01 | TRUE | 1.40E-04 | 49.100 |
| rs10962612 | G | T | 0.014426 | 0.00136487 | 0.761024 | 6.30E-26 | 0.010050 | 0.03 | 0.75 | 7.67E-01 | TRUE | 3.19E-04 | 111.708 |
| rs11070811 | T | C | -0.008823 | 0.00150395 | 0.182873 | 5.10E-09 | 0.048790 | 0.04 | 0.19 | 1.84E-01 | TRUE | 9.82E-05 | 34.413 |
| rs111391498 | G | A | -0.027331 | 0.00271675 | 0.048094 | 6.90E-24 | -0.061875 | 0.07 | 0.04 | 3.84E-01 | TRUE | 2.89E-04 | 101.204 |
| rs11198112 | T | C | -0.016809 | 0.00158946 | 0.165023 | 2.30E-26 | 0.095310 | 0.04 | 0.15 | 1.79E-02 | TRUE | 3.19E-04 | 111.840 |
| rs112089506 | T | C | -0.031302 | 0.00218328 | 0.081295 | 2.00E-47 | 0.086178 | 0.05 | 0.08 | 9.22E-02 | TRUE | 5.87E-04 | 205.554 |
| rs11242899 | A | G | -0.012129 | 0.00131863 | 0.266336 | 1.10E-19 | 0.076961 | 0.03 | 0.28 | 1.02E-02 | TRUE | 2.42E-04 | 84.610 |
| rs11264244 | T | C | -0.011619 | 0.00210281 | 0.085994 | 2.90E-08 | 0.029559 | 0.05 | 0.08 | 6.09E-01 | TRUE | 8.72E-05 | 30.533 |
| rs116125333 | G | T | -0.025883 | 0.00393365 | 0.023117 | 2.50E-10 | 0.095310 | 0.09 | 0.02 | 3.09E-01 | TRUE | 1.24E-04 | 43.295 |
| rs11648436 | T | C | -0.013577 | 0.0012093 | 0.356060 | 7.90E-31 | 0.009950 | 0.03 | 0.35 | 8.11E-01 | TRUE | 3.60E-04 | 126.056 |
| rs117132860 | A | G | 0.033950 | 0.00368208 | 0.025556 | 6.30E-21 | -0.314711 | 0.12 | 0.02 | 8.78E-03 | TRUE | 2.43E-04 | 85.016 |
| rs11739906 | C | A | 0.006903 | 0.0012387 | 0.327118 | 2.70E-08 | 0.000000 | 0.03 | 0.34 | 9.43E-01 | TRUE | 8.87E-05 | 31.057 |
| rs117462393 | T | C | 0.042182 | 0.00519637 | 0.014415 | 5.70E-16 | 0.095310 | 0.11 | 0.02 | 3.70E-01 | TRUE | 1.88E-04 | 65.896 |
| rs12350739 | A | G | 0.018407 | 0.00120001 | 0.607745 | 8.30E-55 | 0.000000 | 0.03 | 0.58 | 9.55E-01 | TRUE | 6.71E-04 | 235.275 |
| rs1267037 | C | A | -0.008423 | 0.00148486 | 0.806239 | 4.10E-08 | -0.009950 | 0.04 | 0.8 | 8.78E-01 | TRUE | 9.19E-05 | 32.176 |
| rs1278766 | C | T | 0.010587 | 0.00117015 | 0.546111 | 2.20E-20 | -0.067659 | 0.03 | 0.54 | 2.21E-02 | TRUE | 2.34E-04 | 81.853 |
| rs12913832 | G | A | 0.034765 | 0.00140463 | 0.777588 | 1.50E-138 | -0.148420 | 0.03 | 0.75 | 3.69E-06 | TRUE | 1.75E-03 | 612.559 |
| rs1308048 | C | T | -0.010936 | 0.00118325 | 0.419442 | 1.00E-19 | 0.019803 | 0.03 | 0.41 | 5.15E-01 | TRUE | 2.44E-04 | 85.425 |
| rs13332673 | T | G | -0.050659 | 0.00579333 | 0.010085 | 1.20E-18 | 0.029559 | 0.14 | 0.01 | 8.57E-01 | TRUE | 2.18E-04 | 76.465 |
| rs1338549 | G | T | -0.007553 | 0.00116776 | 0.532670 | 4.40E-11 | 0.020203 | 0.03 | 0.54 | 5.21E-01 | TRUE | 1.19E-04 | 41.835 |
| rs139414522 | C | T | -0.039621 | 0.00493956 | 0.015298 | 5.30E-16 | 0.139762 | 0.11 | 0.01 | 2.05E-01 | TRUE | 1.84E-04 | 64.339 |
| rs141817469 | T | C | -0.050991 | 0.00317023 | 0.035111 | 2.50E-58 | 0.131028 | 0.08 | 0.03 | 1.04E-01 | TRUE | 7.38E-04 | 258.703 |
| rs142314514 | G | A | -0.043698 | 0.00382382 | 0.028577 | 8.00E-31 | 0.122218 | 0.11 | 0.02 | 2.85E-01 | TRUE | 3.73E-04 | 130.597 |
| rs1437635 | A | C | 0.010176 | 0.00156856 | 0.165792 | 3.60E-11 | -0.010050 | 0.04 | 0.18 | 8.51E-01 | TRUE | 1.20E-04 | 42.089 |
| rs150527451 | A | G | 0.015792 | 0.00189641 | 0.106533 | 2.60E-16 | -0.116534 | 0.05 | 0.12 | 1.33E-02 | TRUE | 1.98E-04 | 69.340 |
| rs1548714 | C | A | 0.008660 | 0.00148979 | 0.811844 | 8.80E-09 | -0.058269 | 0.04 | 0.82 | 1.08E-01 | TRUE | 9.65E-05 | 33.789 |
| rs1584885 | T | G | -0.006184 | 0.00118826 | 0.603707 | 3.40E-08 | 0.030459 | 0.03 | 0.59 | 2.23E-01 | TRUE | 7.73E-05 | 27.088 |
| rs16891982 | G | C | 0.137113 | 0.00358368 | 0.972918 | 1.00E-200 | -0.322083 | 0.06 | 0.96 | 4.75E-07 | TRUE | 4.16E-03 | 1463.849 |
| rs17232484 | A | G | 0.033094 | 0.0049553 | 0.014140 | 4.30E-12 | -0.371064 | 0.16 | 0.01 | 1.70E-02 | TRUE | 1.27E-04 | 44.603 |
| rs2737217 | G | A | -0.014070 | 0.00118004 | 0.562872 | 1.60E-33 | 0.072571 | 0.03 | 0.57 | 1.73E-02 | TRUE | 4.06E-04 | 142.173 |
| rs2793827 | T | A | -0.009696 | 0.00175008 | 0.126104 | 3.60E-08 | 0.067659 | 0.04 | 0.12 | 1.04E-01 | TRUE | 8.76E-05 | 30.697 |
| rs4578351 | C | T | -0.011117 | 0.00140868 | 0.221479 | 2.70E-15 | 0.058269 | 0.03 | 0.22 | 9.86E-02 | TRUE | 1.78E-04 | 62.279 |
| rs4670813 | A | G | -0.007021 | 0.00117199 | 0.470742 | 7.70E-09 | 0.039221 | 0.03 | 0.48 | 1.64E-01 | TRUE | 1.02E-04 | 35.886 |
| rs56360320 | C | A | -0.006904 | 0.00117413 | 0.566674 | 1.40E-09 | -0.039221 | 0.03 | 0.56 | 1.78E-01 | TRUE | 9.87E-05 | 34.575 |
| rs6007506 | T | C | -0.013253 | 0.00123454 | 0.337412 | 7.10E-27 | 0.039221 | 0.03 | 0.34 | 2.11E-01 | TRUE | 3.29E-04 | 115.250 |
| rs61813875 | G | C | 0.022237 | 0.0035909 | 0.030206 | 5.60E-11 | -0.105361 | 0.1 | 0.02 | 3.00E-01 | TRUE | 1.09E-04 | 38.347 |
| rs62423175 | A | G | -0.010229 | 0.00156588 | 0.175551 | 9.50E-11 | 0.000000 | 0.04 | 0.17 | 9.51E-01 | TRUE | 1.22E-04 | 42.676 |
| rs7428430 | T | C | 0.006666 | 0.00116685 | 0.487613 | 1.40E-08 | 0.040822 | 0.03 | 0.51 | 1.51E-01 | TRUE | 9.32E-05 | 32.631 |
| rs75300484 | T | C | -0.017894 | 0.0031568 | 0.034802 | 1.20E-08 | 0.131028 | 0.08 | 0.03 | 9.26E-02 | TRUE | 9.17E-05 | 32.132 |
| rs75459010 | T | C | 0.010577 | 0.0018643 | 0.115487 | 2.90E-08 | -0.030459 | 0.05 | 0.11 | 5.46E-01 | TRUE | 9.19E-05 | 32.185 |
| rs7768317 | T | C | -0.009092 | 0.00134734 | 0.249001 | 2.80E-11 | 0.029559 | 0.03 | 0.24 | 3.78E-01 | TRUE | 1.30E-04 | 45.533 |
| rs7794036 | C | T | -0.007779 | 0.0011654 | 0.530078 | 9.10E-11 | 0.020203 | 0.03 | 0.53 | 5.82E-01 | TRUE | 1.27E-04 | 44.551 |
| rs9328259 | A | C | 0.013462 | 0.00130009 | 0.718160 | 1.80E-25 | -0.076961 | 0.03 | 0.71 | 9.70E-03 | TRUE | 3.06E-04 | 107.223 |
| rs9818780 | C | T | 0.007689 | 0.00116766 | 0.489340 | 2.70E-11 | -0.020203 | 0.03 | 0.48 | 5.09E-01 | TRUE | 1.24E-04 | 43.359 |
| rs9858244 | A | G | 0.009432 | 0.00142087 | 0.214110 | 4.60E-11 | -0.061875 | 0.04 | 0.22 | 5.82E-02 | TRUE | 1.26E-04 | 44.068 |
| rs9926609 | C | A | -0.007435 | 0.00131994 | 0.735838 | 2.50E-08 | 0.020203 | 0.03 | 0.74 | 4.52E-01 | TRUE | 9.06E-05 | 31.733 |
| Childhood sunburn occasions on vitiligo (validation analysis) | | | | | | | | | | | | | |
| rs10168349 | C | G | 0.017342 | 0.00225444 | 0.335704 | 1.40E-14 | 0.000000 | 0.03 | 0.33 | 9.39E-01 | TRUE | 1.71E-04 | 59.175 |
| rs10202908 | T | C | -0.012604 | 0.00227125 | 0.674884 | 2.90E-08 | 0.030459 | 0.03 | 0.69 | 2.76E-01 | TRUE | 8.88E-05 | 30.797 |
| rs10220751 | G | T | -0.013690 | 0.00216389 | 0.404814 | 2.50E-10 | 0.048790 | 0.03 | 0.41 | 1.21E-01 | TRUE | 1.15E-04 | 40.023 |
| rs10788627 | C | T | 0.012019 | 0.00212884 | 0.474528 | 1.60E-08 | -0.040822 | 0.03 | 0.48 | 1.21E-01 | TRUE | 9.19E-05 | 31.873 |
| rs10810636 | G | A | 0.027509 | 0.00249423 | 0.760343 | 2.80E-28 | 0.010050 | 0.03 | 0.75 | 8.37E-01 | TRUE | 3.50E-04 | 121.638 |
| rs10873552 | G | A | 0.012355 | 0.00224357 | 0.657663 | 3.70E-08 | 0.040822 | 0.03 | 0.64 | 1.88E-01 | TRUE | 8.74E-05 | 30.323 |
| rs10896139 | T | C | -0.013634 | 0.00240498 | 0.270905 | 1.40E-08 | 0.039221 | 0.03 | 0.27 | 2.33E-01 | TRUE | 9.26E-05 | 32.138 |
| rs11070811 | T | C | -0.017610 | 0.00274862 | 0.183157 | 1.50E-10 | 0.048790 | 0.04 | 0.19 | 1.84E-01 | TRUE | 1.18E-04 | 41.048 |
| rs111391498 | G | A | -0.047383 | 0.00497472 | 0.047880 | 1.70E-21 | -0.061875 | 0.07 | 0.04 | 3.84E-01 | TRUE | 2.61E-04 | 90.720 |
| rs112089506 | T | C | -0.058086 | 0.00400897 | 0.081196 | 1.40E-47 | 0.086178 | 0.05 | 0.08 | 9.22E-02 | TRUE | 6.05E-04 | 209.929 |
| rs11242899 | A | G | -0.024479 | 0.0024111 | 0.266333 | 3.20E-24 | 0.076961 | 0.03 | 0.28 | 1.02E-02 | TRUE | 2.97E-04 | 103.074 |
| rs116125333 | G | T | -0.039708 | 0.00719516 | 0.023181 | 3.40E-08 | 0.095310 | 0.09 | 0.02 | 3.09E-01 | TRUE | 8.78E-05 | 30.456 |
| rs11648436 | T | C | -0.027274 | 0.00221874 | 0.356354 | 9.90E-35 | 0.009950 | 0.03 | 0.35 | 8.11E-01 | TRUE | 4.35E-04 | 151.104 |
| rs117132860 | A | G | 0.059305 | 0.00672945 | 0.025574 | 1.20E-18 | -0.314711 | 0.12 | 0.02 | 8.78E-03 | TRUE | 2.24E-04 | 77.665 |
| rs11739906 | C | A | 0.013047 | 0.00226464 | 0.327463 | 8.40E-09 | 0.000000 | 0.03 | 0.34 | 9.43E-01 | TRUE | 9.56E-05 | 33.189 |
| rs117462393 | T | C | 0.081482 | 0.00955355 | 0.014374 | 1.50E-17 | 0.095310 | 0.11 | 0.02 | 3.70E-01 | TRUE | 2.10E-04 | 72.743 |
| rs12203592 | T | C | 0.153131 | 0.00252627 | 0.219070 | 1.00E-200 | -0.174353 | 0.04 | 0.14 | 4.49E-05 | TRUE | 1.05E-02 | 3674.212 |
| rs12350739 | A | G | 0.035464 | 0.00219034 | 0.606787 | 5.80E-59 | 0.000000 | 0.03 | 0.58 | 9.55E-01 | TRUE | 7.55E-04 | 262.151 |
| rs1260326 | C | T | -0.012528 | 0.00217252 | 0.603989 | 8.10E-09 | -0.039221 | 0.03 | 0.59 | 2.25E-01 | TRUE | 9.58E-05 | 33.254 |
| rs1267038 | C | A | -0.017889 | 0.00299112 | 0.844933 | 2.20E-09 | 0.010050 | 0.04 | 0.84 | 7.92E-01 | TRUE | 1.03E-04 | 35.768 |
| rs1278766 | C | T | 0.019119 | 0.00213886 | 0.545712 | 3.90E-19 | -0.067659 | 0.03 | 0.54 | 2.21E-02 | TRUE | 2.30E-04 | 79.905 |
| rs12913832 | G | A | 0.060538 | 0.00253089 | 0.775275 | 1.90E-126 | -0.148420 | 0.03 | 0.75 | 3.69E-06 | TRUE | 1.65E-03 | 572.141 |
| rs1308048 | C | T | -0.020350 | 0.00216433 | 0.419538 | 5.30E-21 | 0.019803 | 0.03 | 0.41 | 5.15E-01 | TRUE | 2.55E-04 | 88.406 |
| rs1326798 | G | C | 0.012077 | 0.00219225 | 0.620628 | 3.60E-08 | 0.000000 | 0.03 | 0.61 | 9.49E-01 | TRUE | 8.75E-05 | 30.347 |
| rs13332673 | T | G | -0.102103 | 0.0105348 | 0.010249 | 3.30E-22 | 0.029559 | 0.14 | 0.01 | 8.57E-01 | TRUE | 2.71E-04 | 93.934 |
| rs139414522 | C | T | -0.078388 | 0.00906851 | 0.015298 | 5.40E-18 | 0.139762 | 0.11 | 0.01 | 2.05E-01 | TRUE | 2.15E-04 | 74.719 |
| rs141817469 | T | C | -0.093196 | 0.00581987 | 0.035108 | 1.00E-57 | 0.131028 | 0.08 | 0.03 | 1.04E-01 | TRUE | 7.39E-04 | 256.425 |
| rs142314514 | G | A | -0.081593 | 0.00702727 | 0.028510 | 3.60E-31 | 0.122218 | 0.11 | 0.02 | 2.85E-01 | TRUE | 3.88E-04 | 134.811 |
| rs1437635 | A | C | 0.020699 | 0.00286686 | 0.166029 | 5.20E-13 | -0.010050 | 0.04 | 0.18 | 8.51E-01 | TRUE | 1.50E-04 | 52.127 |
| rs150527451 | A | G | 0.026240 | 0.00347045 | 0.106417 | 4.00E-14 | -0.116534 | 0.05 | 0.12 | 1.33E-02 | TRUE | 1.65E-04 | 57.166 |
| rs151337382 | A | T | 0.057465 | 0.00925005 | 0.013552 | 5.20E-10 | -0.010050 | 0.13 | 0.01 | 9.31E-01 | TRUE | 1.11E-04 | 38.594 |
| rs1548714 | C | A | 0.016158 | 0.00272296 | 0.811733 | 3.00E-09 | -0.058269 | 0.04 | 0.82 | 1.08E-01 | TRUE | 1.01E-04 | 35.212 |
| rs16891982 | G | C | 0.229157 | 0.00620874 | 0.970879 | 1.00E-200 | -0.322083 | 0.06 | 0.96 | 4.75E-07 | TRUE | 3.91E-03 | 1362.250 |
| rs17232484 | A | G | 0.061024 | 0.00907405 | 0.014194 | 1.80E-11 | -0.371064 | 0.16 | 0.01 | 1.70E-02 | TRUE | 1.30E-04 | 45.227 |
| rs1989483 | G | A | 0.012496 | 0.00218968 | 0.389054 | 1.20E-08 | -0.020203 | 0.03 | 0.39 | 4.28E-01 | TRUE | 9.39E-05 | 32.568 |
| rs2299098 | C | G | 0.014791 | 0.00268358 | 0.196234 | 3.60E-08 | -0.051293 | 0.04 | 0.19 | 1.57E-01 | TRUE | 8.76E-05 | 30.379 |
| rs2737217 | G | A | -0.025456 | 0.00215785 | 0.563309 | 4.10E-32 | 0.072571 | 0.03 | 0.57 | 1.73E-02 | TRUE | 4.01E-04 | 139.161 |
| rs35563099 | T | C | -0.031031 | 0.00291491 | 0.164009 | 1.80E-26 | 0.086178 | 0.04 | 0.15 | 3.41E-02 | TRUE | 3.27E-04 | 113.328 |
| rs3759579 | G | A | 0.012449 | 0.00215924 | 0.588839 | 8.10E-09 | 0.000000 | 0.03 | 0.58 | 8.87E-01 | TRUE | 9.58E-05 | 33.241 |
| rs41563 | A | G | 0.013506 | 0.00223217 | 0.349781 | 1.40E-09 | 0.009950 | 0.03 | 0.34 | 7.32E-01 | TRUE | 1.05E-04 | 36.607 |
| rs4240559 | C | T | -0.017105 | 0.00214258 | 0.557906 | 1.40E-15 | 0.030459 | 0.03 | 0.56 | 3.70E-01 | TRUE | 1.84E-04 | 63.732 |
| rs4272574 | T | C | 0.015232 | 0.00212901 | 0.478288 | 8.40E-13 | 0.009950 | 0.03 | 0.5 | 7.03E-01 | TRUE | 1.48E-04 | 51.189 |
| rs4578351 | C | T | -0.020481 | 0.00257235 | 0.222048 | 1.70E-15 | 0.058269 | 0.03 | 0.22 | 9.86E-02 | TRUE | 1.83E-04 | 63.395 |
| rs4670813 | A | G | -0.012669 | 0.00214417 | 0.470775 | 3.50E-09 | 0.039221 | 0.03 | 0.48 | 1.64E-01 | TRUE | 1.01E-04 | 34.910 |
| rs4840542 | T | G | 0.018509 | 0.00213294 | 0.505804 | 4.00E-18 | 0.040822 | 0.03 | 0.51 | 1.51E-01 | TRUE | 2.17E-04 | 75.304 |
| rs537894 | A | G | 0.012726 | 0.00217696 | 0.548976 | 5.00E-09 | -0.019803 | 0.03 | 0.56 | 4.75E-01 | TRUE | 9.85E-05 | 34.171 |
| rs57994353 | C | T | 0.012726 | 0.00232272 | 0.299074 | 4.30E-08 | -0.010050 | 0.03 | 0.3 | 6.74E-01 | TRUE | 8.65E-05 | 30.017 |
| rs6007506 | T | C | -0.024813 | 0.00225726 | 0.337443 | 4.10E-28 | 0.039221 | 0.03 | 0.34 | 2.11E-01 | TRUE | 3.48E-04 | 120.838 |
| rs61816766 | C | T | 0.036578 | 0.00618793 | 0.032281 | 3.40E-09 | -0.040822 | 0.09 | 0.03 | 6.59E-01 | TRUE | 1.01E-04 | 34.942 |
| rs61981034 | A | G | 0.013326 | 0.00243789 | 0.257954 | 4.60E-08 | -0.020203 | 0.03 | 0.27 | 4.59E-01 | TRUE | 8.61E-05 | 29.878 |
| rs62423175 | A | G | -0.018479 | 0.00286453 | 0.175231 | 1.10E-10 | 0.000000 | 0.04 | 0.17 | 9.51E-01 | TRUE | 1.20E-04 | 41.613 |
| rs6882046 | G | A | 0.014350 | 0.00242202 | 0.267930 | 3.10E-09 | -0.061875 | 0.03 | 0.28 | 5.34E-02 | TRUE | 1.01E-04 | 35.103 |
| rs699780 | G | A | -0.018449 | 0.00318541 | 0.127758 | 7.00E-09 | 0.067659 | 0.04 | 0.13 | 1.10E-01 | TRUE | 9.67E-05 | 33.542 |
| rs72821630 | T | C | -0.015041 | 0.00245522 | 0.251407 | 9.00E-10 | -0.010050 | 0.03 | 0.25 | 7.51E-01 | TRUE | 1.08E-04 | 37.527 |
| rs75300484 | T | C | -0.034208 | 0.00580191 | 0.034690 | 3.70E-09 | 0.131028 | 0.08 | 0.03 | 9.26E-02 | TRUE | 1.00E-04 | 34.763 |
| rs75908072 | C | T | -0.031759 | 0.00571651 | 0.036612 | 2.80E-08 | 0.039221 | 0.08 | 0.03 | 6.12E-01 | TRUE | 8.90E-05 | 30.865 |
| rs7768317 | T | C | -0.017032 | 0.00246523 | 0.248878 | 4.90E-12 | 0.029559 | 0.03 | 0.24 | 3.78E-01 | TRUE | 1.38E-04 | 47.730 |
| rs784235 | G | A | -0.016385 | 0.0027625 | 0.819090 | 3.00E-09 | 0.000000 | 0.04 | 0.82 | 9.71E-01 | TRUE | 1.01E-04 | 35.179 |
| rs78444298 | A | G | -0.046672 | 0.00771154 | 0.019692 | 1.40E-09 | -0.030459 | 0.11 | 0.02 | 8.09E-01 | TRUE | 1.06E-04 | 36.629 |
| rs9328259 | A | C | 0.026744 | 0.00237012 | 0.718185 | 1.60E-29 | -0.076961 | 0.03 | 0.71 | 9.70E-03 | TRUE | 3.67E-04 | 127.323 |
| rs964127 | T | A | 0.012307 | 0.00225692 | 0.332818 | 4.90E-08 | 0.009950 | 0.03 | 0.34 | 7.11E-01 | TRUE | 8.57E-05 | 29.736 |
| rs9821675 | G | A | -0.012037 | 0.00212501 | 0.505270 | 1.50E-08 | -0.010050 | 0.03 | 0.49 | 7.30E-01 | TRUE | 9.25E-05 | 32.087 |
| rs9832130 | A | G | -0.012794 | 0.00216232 | 0.580680 | 3.30E-09 | -0.048790 | 0.03 | 0.57 | 7.77E-02 | TRUE | 1.01E-04 | 35.010 |
| rs9858244 | A | G | 0.018945 | 0.00259673 | 0.214182 | 3.00E-13 | -0.061875 | 0.04 | 0.22 | 5.82E-02 | TRUE | 1.53E-04 | 53.225 |
| rs9867857 | T | C | 0.013625 | 0.00213562 | 0.489160 | 1.80E-10 | -0.020203 | 0.03 | 0.48 | 5.03E-01 | TRUE | 1.17E-04 | 40.701 |
| Ease of skin tanning on vitiligo (validation analysis) | | | | | | | | | | | | | |
| rs10054039 | A | G | -0.012765 | 0.00207698 | 0.373657 | 8.00E-10 | 0.000000 | 0.03 | 0.39 | 9.58E-01 | TRUE | 8.34E-05 | 37.771 |
| rs10202360 | C | A | -0.021801 | 0.00262681 | 0.177348 | 1.00E-16 | 0.009950 | 0.04 | 0.18 | 7.35E-01 | TRUE | 1.52E-04 | 68.881 |
| rs1031896 | C | T | -0.011944 | 0.00207686 | 0.382321 | 8.90E-09 | 0.000000 | 0.03 | 0.38 | 9.35E-01 | TRUE | 7.30E-05 | 33.075 |
| rs1046793 | T | C | 0.037606 | 0.00201029 | 0.537961 | 4.40E-78 | -0.067659 | 0.03 | 0.53 | 2.02E-02 | TRUE | 7.72E-04 | 349.945 |
| rs10794666 | T | C | 0.015740 | 0.00202365 | 0.572003 | 7.40E-15 | -0.048790 | 0.03 | 0.55 | 6.92E-02 | TRUE | 1.34E-04 | 60.495 |
| rs10810636 | G | A | 0.056176 | 0.00235127 | 0.760701 | 3.70E-126 | 0.010050 | 0.03 | 0.75 | 8.37E-01 | TRUE | 1.26E-03 | 570.806 |
| rs10879095 | A | G | -0.013180 | 0.00208683 | 0.639015 | 2.70E-10 | 0.040822 | 0.03 | 0.63 | 1.43E-01 | TRUE | 8.80E-05 | 39.891 |
| rs10897156 | A | T | 0.095083 | 0.0120683 | 0.993027 | 3.30E-15 | -0.067659 | 0.12 | 0.99 | 5.84E-01 | TRUE | 1.37E-04 | 62.075 |
| rs10947996 | T | G | -0.018736 | 0.00230495 | 0.251254 | 4.30E-16 | 0.039221 | 0.03 | 0.25 | 2.25E-01 | TRUE | 1.46E-04 | 66.075 |
| rs11070811 | T | C | -0.029807 | 0.00258887 | 0.182822 | 1.10E-30 | 0.048790 | 0.04 | 0.19 | 1.84E-01 | TRUE | 2.93E-04 | 132.562 |
| rs11104947 | A | G | 0.118269 | 0.00822546 | 0.015194 | 7.10E-47 | -0.385662 | 0.15 | 0.01 | 1.15E-02 | TRUE | 4.56E-04 | 206.738 |
| rs111310469 | T | C | 0.045452 | 0.00761264 | 0.018410 | 2.40E-09 | 0.122218 | 0.1 | 0.02 | 2.12E-01 | TRUE | 7.87E-05 | 35.648 |
| rs112089506 | T | C | -0.137978 | 0.00373465 | 0.080846 | 1.00E-200 | 0.086178 | 0.05 | 0.08 | 9.22E-02 | TRUE | 3.00E-03 | 1364.954 |
| rs112217744 | A | G | -0.035499 | 0.00489277 | 0.043784 | 4.00E-13 | -0.010050 | 0.07 | 0.04 | 9.13E-01 | TRUE | 1.16E-04 | 52.640 |
| rs1123483 | G | A | 0.015598 | 0.00211047 | 0.657258 | 1.50E-13 | -0.067659 | 0.03 | 0.66 | 2.24E-02 | TRUE | 1.21E-04 | 54.622 |
| rs11242899 | A | G | -0.033873 | 0.0022686 | 0.266624 | 2.10E-50 | 0.076961 | 0.03 | 0.28 | 1.02E-02 | TRUE | 4.92E-04 | 222.939 |
| rs1129614 | A | G | -0.016602 | 0.0024445 | 0.214257 | 1.10E-11 | 0.048790 | 0.03 | 0.21 | 1.30E-01 | TRUE | 1.02E-04 | 46.126 |
| rs113151897 | T | A | -0.021676 | 0.00372063 | 0.080142 | 5.70E-09 | 0.019803 | 0.05 | 0.08 | 7.19E-01 | TRUE | 7.49E-05 | 33.939 |
| rs1137134 | A | G | 0.043611 | 0.00204749 | 0.602148 | 1.10E-100 | -0.009950 | 0.03 | 0.59 | 8.35E-01 | TRUE | 1.00E-03 | 453.679 |
| rs117132860 | A | G | 0.148572 | 0.0063036 | 0.025972 | 7.91E-123 | -0.314711 | 0.12 | 0.02 | 8.78E-03 | TRUE | 1.22E-03 | 555.514 |
| rs11907438 | A | T | -0.052101 | 0.00361982 | 0.083291 | 5.70E-47 | 0.029559 | 0.05 | 0.1 | 5.89E-01 | TRUE | 4.57E-04 | 207.162 |
| rs11954036 | C | T | 0.023666 | 0.00213796 | 0.326762 | 1.80E-28 | 0.000000 | 0.03 | 0.34 | 9.92E-01 | TRUE | 2.70E-04 | 122.530 |
| rs12146565 | A | G | 0.013887 | 0.00214204 | 0.321425 | 9.00E-11 | -0.020203 | 0.03 | 0.33 | 4.97E-01 | TRUE | 9.28E-05 | 42.029 |
| rs12350739 | A | G | 0.074071 | 0.00206609 | 0.607970 | 1.00E-200 | 0.000000 | 0.03 | 0.58 | 9.55E-01 | TRUE | 2.83E-03 | 1285.261 |
| rs12550943 | A | G | 0.012409 | 0.00222058 | 0.289927 | 2.30E-08 | -0.040822 | 0.03 | 0.29 | 2.41E-01 | TRUE | 6.89E-05 | 31.229 |
| rs12913832 | G | A | 0.154261 | 0.00239043 | 0.777053 | 1.00E-200 | -0.148420 | 0.03 | 0.75 | 3.69E-06 | TRUE | 9.11E-03 | 4164.456 |
| rs137879272 | A | C | -0.201986 | 0.00897314 | 0.014126 | 3.30E-112 | 0.343590 | 0.08 | 0.02 | 2.63E-05 | TRUE | 1.12E-03 | 506.701 |
| rs141817469 | T | C | -0.177358 | 0.00542639 | 0.034919 | 1.00E-200 | 0.131028 | 0.08 | 0.03 | 1.04E-01 | TRUE | 2.35E-03 | 1068.262 |
| rs142314514 | G | A | -0.185300 | 0.00655734 | 0.028340 | 1.10E-175 | 0.122218 | 0.11 | 0.02 | 2.85E-01 | TRUE | 1.76E-03 | 798.534 |
| rs146048597 | A | G | -0.017282 | 0.00300238 | 0.123531 | 8.60E-09 | 0.048790 | 0.04 | 0.12 | 2.14E-01 | TRUE | 7.31E-05 | 33.131 |
| rs147535976 | A | G | -0.100712 | 0.0122362 | 0.006870 | 1.90E-16 | -0.235722 | 0.13 | 0.01 | 7.17E-02 | TRUE | 1.50E-04 | 67.743 |
| rs1483734 | C | T | -0.013418 | 0.00238304 | 0.233494 | 1.80E-08 | 0.009950 | 0.03 | 0.24 | 7.80E-01 | TRUE | 7.00E-05 | 31.705 |
| rs1493698 | C | T | -0.018357 | 0.00304262 | 0.124181 | 1.60E-09 | 0.067659 | 0.04 | 0.12 | 1.19E-01 | TRUE | 8.03E-05 | 36.401 |
| rs1613999 | G | T | -0.034218 | 0.00203939 | 0.421009 | 3.50E-63 | 0.019803 | 0.03 | 0.42 | 5.53E-01 | TRUE | 6.21E-04 | 281.525 |
| rs16891982 | G | C | 0.406336 | 0.00600135 | 0.972206 | 1.00E-200 | -0.322083 | 0.06 | 0.96 | 4.75E-07 | TRUE | 1.00E-02 | 4584.276 |
| rs1721040 | A | T | 0.024611 | 0.00204234 | 0.417781 | 1.90E-33 | -0.030459 | 0.03 | 0.42 | 3.66E-01 | TRUE | 3.20E-04 | 145.216 |
| rs17232484 | A | G | 0.123729 | 0.00841391 | 0.014260 | 6.00E-49 | -0.371064 | 0.16 | 0.01 | 1.70E-02 | TRUE | 4.77E-04 | 216.245 |
| rs177114 | C | A | 0.011353 | 0.00200784 | 0.524990 | 1.60E-08 | -0.019803 | 0.03 | 0.53 | 6.13E-01 | TRUE | 7.06E-05 | 31.969 |
| rs1800440 | C | T | -0.034060 | 0.00257326 | 0.186036 | 5.40E-40 | 0.029559 | 0.04 | 0.19 | 4.26E-01 | TRUE | 3.87E-04 | 175.196 |
| rs1805007 | T | C | 0.427724 | 0.00323332 | 0.102727 | 1.00E-200 | -0.597837 | 0.07 | 0.08 | 5.67E-19 | TRUE | 3.72E-02 | 17499.593 |
| rs1805008 | T | C | 0.305776 | 0.0034858 | 0.087531 | 1.00E-200 | -0.430783 | 0.06 | 0.08 | 7.16E-12 | TRUE | 1.67E-02 | 7694.846 |
| rs1870940 | A | G | 0.020411 | 0.0022705 | 0.271365 | 2.50E-19 | 0.000000 | 0.03 | 0.26 | 9.31E-01 | TRUE | 1.78E-04 | 80.815 |
| rs1885194 | C | T | 0.035229 | 0.00201714 | 0.448275 | 2.70E-68 | -0.061875 | 0.03 | 0.44 | 3.06E-02 | TRUE | 6.73E-04 | 305.019 |
| rs2167957 | T | C | 0.017282 | 0.00231149 | 0.745779 | 7.60E-14 | -0.048790 | 0.03 | 0.74 | 1.19E-01 | TRUE | 1.23E-04 | 55.900 |
| rs2236295 | T | G | -0.015876 | 0.00205345 | 0.403189 | 1.10E-14 | -0.051293 | 0.03 | 0.39 | 1.17E-01 | TRUE | 1.32E-04 | 59.771 |
| rs2275657 | C | G | -0.016057 | 0.00208155 | 0.367001 | 1.20E-14 | 0.058269 | 0.03 | 0.38 | 3.34E-02 | TRUE | 1.31E-04 | 59.501 |
| rs2737207 | A | C | -0.044273 | 0.00202675 | 0.556779 | 8.79E-106 | 0.072571 | 0.03 | 0.57 | 1.27E-02 | TRUE | 1.05E-03 | 477.166 |
| rs308896 | T | G | -0.075160 | 0.00750597 | 0.018457 | 1.30E-23 | 0.239017 | 0.09 | 0.02 | 8.78E-03 | TRUE | 2.21E-04 | 100.266 |
| rs329120 | T | C | 0.011380 | 0.00203449 | 0.419224 | 2.20E-08 | -0.020203 | 0.03 | 0.42 | 4.43E-01 | TRUE | 6.91E-05 | 31.289 |
| rs34635363 | A | G | 0.023500 | 0.00209302 | 0.358436 | 3.00E-29 | 0.000000 | 0.03 | 0.36 | 9.63E-01 | TRUE | 2.78E-04 | 126.061 |
| rs35380972 | C | A | -0.020656 | 0.00208286 | 0.368172 | 3.50E-23 | 0.048790 | 0.03 | 0.37 | 1.12E-01 | TRUE | 2.17E-04 | 98.345 |
| rs3743538 | T | G | -0.016591 | 0.00206483 | 0.352448 | 9.40E-16 | 0.019803 | 0.03 | 0.35 | 5.38E-01 | TRUE | 1.42E-04 | 64.562 |
| rs3851294 | G | A | -0.047498 | 0.00345627 | 0.907723 | 5.70E-43 | 0.072571 | 0.05 | 0.9 | 1.60E-01 | TRUE | 4.17E-04 | 188.853 |
| rs399856 | A | G | -0.015868 | 0.00207445 | 0.620560 | 2.00E-14 | -0.019803 | 0.03 | 0.62 | 5.53E-01 | TRUE | 1.29E-04 | 58.507 |
| rs4257287 | T | C | -0.018601 | 0.00319856 | 0.887895 | 6.00E-09 | 0.010050 | 0.05 | 0.9 | 7.71E-01 | TRUE | 7.46E-05 | 33.819 |
| rs4406278 | T | G | 0.042633 | 0.00222878 | 0.718260 | 1.50E-81 | -0.076961 | 0.03 | 0.71 | 9.90E-03 | TRUE | 8.07E-04 | 365.898 |
| rs4592422 | T | C | 0.066607 | 0.00343362 | 0.906242 | 8.00E-84 | -0.122218 | 0.05 | 0.91 | 9.56E-03 | TRUE | 8.30E-04 | 376.295 |
| rs4689314 | C | T | 0.023449 | 0.00407746 | 0.935179 | 8.90E-09 | -0.076961 | 0.06 | 0.94 | 1.92E-01 | TRUE | 7.30E-05 | 33.073 |
| rs4756838 | T | C | 0.016558 | 0.00269821 | 0.167791 | 8.40E-10 | -0.020203 | 0.04 | 0.15 | 5.82E-01 | TRUE | 8.31E-05 | 37.656 |
| rs4778984 | G | A | 0.015267 | 0.00228915 | 0.256877 | 2.60E-11 | -0.061875 | 0.03 | 0.26 | 5.05E-02 | TRUE | 9.82E-05 | 44.476 |
| rs4788566 | C | T | 0.018817 | 0.0022298 | 0.732878 | 3.20E-17 | -0.058269 | 0.03 | 0.73 | 9.29E-02 | TRUE | 1.57E-04 | 71.217 |
| rs4933379 | T | C | 0.012754 | 0.00201455 | 0.468545 | 2.40E-10 | -0.051293 | 0.03 | 0.47 | 5.49E-02 | TRUE | 8.85E-05 | 40.081 |
| rs4939832 | G | A | -0.013217 | 0.00235351 | 0.242885 | 2.00E-08 | -0.040822 | 0.03 | 0.24 | 2.15E-01 | TRUE | 6.96E-05 | 31.538 |
| rs6007506 | T | C | -0.036322 | 0.00213106 | 0.336835 | 3.90E-65 | 0.039221 | 0.03 | 0.34 | 2.11E-01 | TRUE | 6.41E-04 | 290.503 |
| rs60143966 | A | C | -0.015854 | 0.00237852 | 0.239145 | 2.60E-11 | 0.000000 | 0.03 | 0.24 | 8.94E-01 | TRUE | 9.80E-05 | 44.426 |
| rs6059013 | A | G | -0.016798 | 0.00234264 | 0.240526 | 7.50E-13 | 0.029559 | 0.03 | 0.24 | 3.96E-01 | TRUE | 1.13E-04 | 51.415 |
| rs6059655 | G | A | -0.273292 | 0.0033504 | 0.896917 | 1.00E-200 | 0.462035 | 0.06 | 0.92 | 3.58E-13 | TRUE | 1.45E-02 | 6653.629 |
| rs6061182 | C | T | -0.011362 | 0.00201668 | 0.562875 | 1.80E-08 | -0.029559 | 0.03 | 0.57 | 3.51E-01 | TRUE | 7.01E-05 | 31.742 |
| rs60780889 | T | C | -0.020057 | 0.00228627 | 0.259227 | 1.70E-18 | 0.067659 | 0.03 | 0.28 | 3.91E-02 | TRUE | 1.70E-04 | 76.959 |
| rs6088232 | T | C | -0.062364 | 0.00784196 | 0.017951 | 1.80E-15 | 0.039221 | 0.13 | 0.01 | 7.72E-01 | TRUE | 1.40E-04 | 63.244 |
| rs61348812 | C | T | 0.014761 | 0.00256965 | 0.811921 | 9.20E-09 | -0.058269 | 0.04 | 0.82 | 9.90E-02 | TRUE | 7.28E-05 | 32.999 |
| rs61935849 | C | A | 0.027523 | 0.00318467 | 0.111948 | 5.50E-18 | -0.010050 | 0.05 | 0.11 | 9.10E-01 | TRUE | 1.65E-04 | 74.691 |
| rs62435865 | C | T | 0.021432 | 0.00316189 | 0.115279 | 1.20E-11 | -0.030459 | 0.05 | 0.12 | 5.04E-01 | TRUE | 1.01E-04 | 45.945 |
| rs6681924 | G | A | -0.013053 | 0.00231628 | 0.750657 | 1.70E-08 | 0.000000 | 0.03 | 0.75 | 9.58E-01 | TRUE | 7.01E-05 | 31.756 |
| rs670318 | C | T | -0.046988 | 0.00466649 | 0.951699 | 7.60E-24 | 0.105361 | 0.07 | 0.96 | 1.75E-01 | TRUE | 2.24E-04 | 101.391 |
| rs6790699 | G | A | 0.019593 | 0.00207249 | 0.626661 | 3.30E-21 | 0.072571 | 0.03 | 0.62 | 1.09E-02 | TRUE | 1.97E-04 | 89.372 |
| rs68099344 | T | A | 0.018704 | 0.00264455 | 0.174310 | 1.50E-12 | -0.030459 | 0.04 | 0.17 | 3.70E-01 | TRUE | 1.10E-04 | 50.021 |
| rs68173807 | G | A | 0.012882 | 0.0021372 | 0.329079 | 1.70E-09 | 0.009950 | 0.03 | 0.32 | 6.99E-01 | TRUE | 8.02E-05 | 36.328 |
| rs6879874 | T | A | -0.015389 | 0.00224268 | 0.723617 | 6.80E-12 | -0.029559 | 0.03 | 0.74 | 3.17E-01 | TRUE | 1.04E-04 | 47.083 |
| rs6914598 | C | T | 0.014839 | 0.00215256 | 0.323950 | 5.40E-12 | 0.058269 | 0.03 | 0.31 | 4.80E-02 | TRUE | 1.05E-04 | 47.523 |
| rs7098111 | T | C | -0.056163 | 0.00275585 | 0.163162 | 2.50E-92 | 0.076961 | 0.04 | 0.15 | 3.72E-02 | TRUE | 9.16E-04 | 415.326 |
| rs7249850 | A | G | -0.011162 | 0.00203248 | 0.428022 | 4.00E-08 | 0.000000 | 0.03 | 0.44 | 9.11E-01 | TRUE | 6.66E-05 | 30.158 |
| rs72632979 | G | A | -0.022156 | 0.00265995 | 0.172279 | 8.10E-17 | 0.076961 | 0.04 | 0.17 | 5.36E-02 | TRUE | 1.53E-04 | 69.378 |
| rs726558 | G | A | -0.012957 | 0.00226045 | 0.298149 | 9.90E-09 | -0.030459 | 0.03 | 0.3 | 2.81E-01 | TRUE | 7.25E-05 | 32.857 |
| rs72708989 | T | C | 0.037140 | 0.00510302 | 0.040109 | 3.40E-13 | -0.094311 | 0.09 | 0.03 | 2.99E-01 | TRUE | 1.17E-04 | 52.969 |
| rs72710504 | T | C | 0.056578 | 0.0076507 | 0.017589 | 1.40E-13 | -0.061875 | 0.11 | 0.02 | 5.69E-01 | TRUE | 1.21E-04 | 54.688 |
| rs72917317 | G | T | 0.085858 | 0.0032533 | 0.107146 | 1.70E-153 | -0.116534 | 0.05 | 0.12 | 1.35E-02 | TRUE | 1.53E-03 | 696.478 |
| rs72954723 | A | G | -0.040519 | 0.00652817 | 0.024086 | 5.40E-10 | -0.261365 | 0.16 | 0.01 | 9.46E-02 | TRUE | 8.50E-05 | 38.524 |
| rs73087086 | G | A | 0.015145 | 0.00239228 | 0.227840 | 2.40E-10 | -0.020203 | 0.03 | 0.22 | 6.25E-01 | TRUE | 8.85E-05 | 40.079 |
| rs75223371 | A | G | -0.034140 | 0.00485554 | 0.044727 | 2.00E-12 | 0.095310 | 0.07 | 0.04 | 1.52E-01 | TRUE | 1.09E-04 | 49.435 |
| rs75300484 | T | C | -0.055748 | 0.00540312 | 0.034584 | 5.90E-25 | 0.131028 | 0.08 | 0.03 | 9.26E-02 | TRUE | 2.35E-04 | 106.455 |
| rs75575928 | T | C | 0.014042 | 0.00238855 | 0.232603 | 4.10E-09 | -0.020203 | 0.03 | 0.23 | 5.60E-01 | TRUE | 7.63E-05 | 34.563 |
| rs7589217 | G | A | -0.011250 | 0.00204715 | 0.399191 | 3.90E-08 | -0.030459 | 0.03 | 0.39 | 3.76E-01 | TRUE | 6.67E-05 | 30.202 |
| rs75908072 | C | T | -0.047239 | 0.00539862 | 0.036302 | 2.10E-18 | 0.039221 | 0.08 | 0.03 | 6.12E-01 | TRUE | 1.69E-04 | 76.564 |
| rs77568325 | G | A | -0.040219 | 0.00643072 | 0.025035 | 4.00E-10 | 0.148420 | 0.09 | 0.02 | 8.85E-02 | TRUE | 8.63E-05 | 39.114 |
| rs77580397 | T | C | 0.013791 | 0.00248629 | 0.205120 | 2.90E-08 | -0.020203 | 0.04 | 0.2 | 6.57E-01 | TRUE | 6.79E-05 | 30.766 |
| rs8007832 | C | T | 0.011813 | 0.00209844 | 0.363861 | 1.80E-08 | 0.009950 | 0.03 | 0.36 | 8.02E-01 | TRUE | 6.99E-05 | 31.691 |
| rs8023038 | A | G | 0.011990 | 0.00213481 | 0.656699 | 2.00E-08 | 0.040822 | 0.03 | 0.64 | 1.43E-01 | TRUE | 6.96E-05 | 31.544 |
| rs8028550 | C | T | 0.012368 | 0.00204769 | 0.599338 | 1.50E-09 | 0.010050 | 0.03 | 0.6 | 6.56E-01 | TRUE | 8.05E-05 | 36.481 |
| rs8182578 | A | G | 0.011260 | 0.00203916 | 0.430283 | 3.40E-08 | 0.019803 | 0.03 | 0.43 | 4.89E-01 | TRUE | 6.73E-05 | 30.493 |
| rs849138 | A | G | 0.017948 | 0.00200889 | 0.507044 | 4.10E-19 | -0.076961 | 0.03 | 0.5 | 9.57E-03 | TRUE | 1.76E-04 | 79.824 |
| rs899381 | C | T | -0.070663 | 0.00968893 | 0.010992 | 3.00E-13 | -0.174353 | 0.14 | 0.01 | 2.17E-01 | TRUE | 1.17E-04 | 53.190 |
| rs9302151 | C | T | -0.011835 | 0.00200283 | 0.528038 | 3.40E-09 | 0.020203 | 0.03 | 0.54 | 5.37E-01 | TRUE | 7.71E-05 | 34.915 |
| rs9561570 | T | G | 0.021589 | 0.0021626 | 0.314884 | 1.80E-23 | 0.009950 | 0.03 | 0.32 | 7.42E-01 | TRUE | 2.20E-04 | 99.659 |
| rs9821965 | G | A | -0.012202 | 0.00204961 | 0.396818 | 2.60E-09 | -0.010050 | 0.03 | 0.4 | 6.74E-01 | TRUE | 7.82E-05 | 35.441 |
| rs9867857 | T | C | 0.023701 | 0.00201471 | 0.489741 | 6.00E-32 | -0.020203 | 0.03 | 0.48 | 5.03E-01 | TRUE | 3.05E-04 | 138.387 |
